# Characteristics of successful programmes targeting gender inequality and restrictive gender norms for the health and wellbeing of children, adolescents, and young adults: a systematic review

**DOI:** 10.1016/S2214-109X(19)30495-4

**Published:** 2019-12-23

**Authors:** Jessica K Levy, Gary L Darmstadt, Caitlin Ashby, Mary Quandt, Erika Halsey, Aishwarya Nagar, Margaret E Greene

**Affiliations:** a Brown School at Washington University in St Louis, St Louis, MO, USA; b Iris Group, Chapel Hill, NC, USA; c Department of Pediatrics, Stanford University School of Medicine, Palo Alto, CA, USA; d GreeneWorks, Washington, DC, USA

## Abstract

**Background:**

In the context of the Sustainable Development Goals and the shifting global burden of disease, this systematic review analyses the evidence from rigorously evaluated programmes that seek to transform the gendered social norms undermining the health and wellbeing of children, adolescents, and young adults. The aim of this study was threefold: to describe the landscape of gender-transformative programmes that attempt to influence health-related outcomes; to identify mechanisms through which successful programmes work; and to highlight where gaps might exist in implementation and evaluation.

**Methods:**

We systematically reviewed rigorous evaluations published between Jan 1, 2000, and Nov 1, 2018 of programmes that sought to decrease gender inequalities and transform restrictive gender norms to improve the health and wellbeing of 0–24 year olds. We included rigorously evaluated health programmes that met the Interagency Gender Working Group definition of gender-transformative programming, regardless of where in the world they were implemented and what area of health they focused on.

**Findings:**

Among 22 993 articles identified by our search, 61 evaluations of 59 programmes met review criteria. Programmes were concentrated in sub-Saharan Africa (25 [42%]), south Asia (13 [22%]), and North America (13 [22%]) and mainly measured health indicators related to reproductive health (29 [48%]), violence (26 [43%]), or HIV (18 [30%]). Programmes most frequently focused on improving the individual power of the beneficiaries, rather than working on broader systems of inequality. 45 (74%) of the evaluations measured significant improvements in health-related and gender-related indicators; however, only ten (16%) showed evidence of, or potential for, broader norm change. These ten programmes worked with sectors beyond health, included multiple stakeholders, implemented diversified strategies, and fostered critical awareness and participation among affected community members.

**Interpretation:**

This review can accelerate efforts to improve global health by leading to more strategic investment in programmes that promote gender equality and target restrictive gender norms among young people. Such programmes can lead to a lifetime of improved health and wellbeing by challenging not only attitudes and behaviours related to gender at an early age, but also the gendered systems that surround them.

**Funding:**

Bill & Melinda Gates Foundation.

## Introduction

The health consequences of gender inequality have the greatest effect on women and girls, but restrictive gender norms harm health and limit life choices for all.^1^, ^2^ Gender norms are embedded in community culture and institutions^3^ as the often-unspoken rules that govern beliefs about how individuals in the community should behave (ie, injunctive norms, beliefs about what should be) and perceptions of what most people in the community actually do (ie, descriptive norms, empirical beliefs). For example, normative expressions of masculinity often require men to be tough and stoic, proving their manliness through participation in risky behaviours;^4^ whereas normative expressions of femininity encourage women's sexual passivity and deference to male partners for important health-related decisions.^5^ Additionally, gender minorities are subject to ostracism and poor quality or even abusive health care for not conforming to a strict gender binary.^6^

Depending on their gender, children, adolescents, and young adults are valued differently within society and face divergent expectations, leading to potentially harmful customs, such as child marriage for girls and risky forms of labour for boys.^7^ These values, along with discriminatory apportioning of resources and power, are often reinforced by parents, families, peers, and the media.^8^ In infancy and early childhood, restrictive gender norms might lead to unequal breastfeeding or immunisation practices,^9^ differential health-care seeking, or the proffering of gendered toys that stimulate disparate spatial reasoning skills.^10^ Building on these patterns, gender inequality and restrictive norms are strongly reinforced and internalised during adolescence^11^ when key transitions to individualised identities, sexual activity, labour force participation, and marriage also take place.^12^ Patterns of behaviour that can last a lifetime are established during adolescence, offering an opportunity to prevent gendered health inequities by intervening at an early stage.

**Research in context**

**Evidence before this study**

This analysis builds on and brings together two previous streams of research. The first stream is comprised of systematic reviews of the evidence for the effect of gender-transformative interventions on sexual and reproductive health, HIV, and violence outcomes in low-income and middle-income countries; the second stream identifies individual, interpersonal, and community-level predictors of gender norms among young adolescents. In the context of meeting the Sustainable Development Goals and responding to the shifting global burden of disease, this systematic review highlights what is being done programmatically to address the influence of gender inequality and restrictive gender norms on child and adolescent health.

**Added value of this study**

This systematic review builds on existing literature reviews by searching for and including evaluations of any type of programme, in any geographical location, that intentionally addressed gender norms and power imbalances (ie, sought to be gender transformative) to benefit 0–24 year olds, and rigorously measured potential changes in health-related outcomes. We found that three-quarters of all programmes that met our study criteria significantly impacted both gender-related and health-related variables simultaneously. The highest quality programmes shared several characteristics, which suggest that they have potential to powerfully affect both gender equality and health. These programmes worked with sectors beyond health to create change; included the participation of multiple stakeholders; implemented diversified strategies; and fostered critical awareness and participation among affected community members, encouraging them to become active agents in shaping their own health.

**Implications of all the available evidence**

Our research highlights gaps in implementation of gender-transformative activities, including the types of activities being implemented and the underlying norms and health outcomes that interventions have focused on. The shift in the global burden of disease among young people calls for a sharper focus on how to shift the gender-related norms that contribute to poor mental health, infectious disease, substance use, injury, and chronic disease. Our focus on children, adolescents, and young adults highlights the global opportunity to address gendered constraints on health and wellbeing at an early age to ensure young people benefit from the effects for their lifetime.

Given the importance of this age group, this systematic review aims to supplement the *Lancet* Series on Gender Norms and Health.^13^ The review takes a deeper look at rigorously evaluated programmes that sought to decrease gender inequalities and transform restrictive gender norms among 0–24 year-olds to describe the landscape of existing programmes and identify gaps in implementation, as well as highlight methods that work. This systematic review analyses evidence from rigorously evaluated programmes that sought to decrease gender inequalities and transform restrictive gender norms to improve the health and wellbeing of young people. It comes at a time when practitioners are looking for new ways to address the shifting global burden of disease among children, adolescents, and young adults, and to meet the Sustainable Development Goals, one of which is to achieve gender equality.^14^ Building on existing literature reviews that largely focused on programmes implemented in low-income and middle-income countries and measured sexual and reproductive health outcomes,^15^, ^16^ this review offers insights for programmes addressing any health outcome in any part of the world. We aimed to identify mechanisms through which successful programmes work, and where there might be gaps in implementation and evaluation.

## Methods

### Search strategy and selection criteria

We did a systematic review of the peer-reviewed literature and a comprehensive review of the grey literature following PRISMA guidelines (appendix pp 1–2) to identify health programmes with so-called gender transformative intent that focused on children, adolescents, and young adults up to age 24 years. We included only programmes that rigorously measured health-related outcomes and that met at least one of the widely used criteria of the Interagency Gender Working Group's definition of gender-transformative programming: “programmes that seek to transform gender relations to promote equality and achieve programme objectives…by (1) fostering critical examination of inequalities and gender roles, norms, and dynamics; (2) recognising and strengthening positive norms that support equality and an enabling environment; (3) promoting the relative position of women, girls, and marginalised groups; and (4) transforming the underlying social structures, policies, and broadly held social norms that perpetuate gender inequalities”.^17^ In keeping with the Interagency Gender Working Group's definition, all programmes included in our systematic review at a minimum had a stated aim of transforming participants' attitudes, beliefs, or behaviours related to the restrictive gender norms in their settings, and at best, hoped to use such transformations to challenge and even shift gender norms overall.

We reviewed evaluations published in English, French, Spanish, or Portuguese between Jan 1, 2000, and Nov 1, 2018, that used quantitative, qualitative, or mixed methods to infer a high level of causality. To optimise the quality of studies included in the review, we adapted a protocol used by the Overseas Development Institute^18^ that used the Mixed Methods Appraisal Tool to assess quality of study design.^19^ For quantitative evaluations, either randomised controlled trials (RCTs) or quasi-experimental designs were required, each with a participant retention of at least 60% and a sample size of at least 50 people per experimental group for RCTs and 100 people per experimental group for quasi-experimental designs.^18^ Evaluations using qualitative methods must have interviewed at least 30 individuals or held at least six focus groups, with two or more coders to analyse and cross-check data.^20^, ^21^ Mixed-methods studies were included if either their quantitative or qualitative portion met the criteria we have described (appendix pp 3–4).

We ran our search terms (appendix pp 5–6) in Scopus, EBSCO, and Web of Science. Our search included terms that referenced age, programme, evaluation type, and gender-transformative intent. After eliminating duplicates, we screened titles and abstracts, and did a secondary screening of full-text articles on the basis of our inclusion criteria. Using the same terms, we also did a comprehensive internet search of the grey literature and a targeted search of 17 organisations that work on health and gender (appendix p 7).

### Data analysis

Two researchers independently coded and categorised key programmatic material, using the software programme EPPI-Reviewer (appendix pp 8–14); differences were reconciled by a third study team member. We then used descriptive statistics to identify patterns in programme design and implementation, as well as themes that emerged among programmes that produced statistically significant outcomes related to gender or health. Quantitative and qualitative data were analysed using the same coding technique. Consistent with methods described by Heymann and colleagues,^13^ we conducted an additional level of analysis to identify high-quality programmes that showed four overlapping criteria associated with broader norm change: multiplicity (improvement in outcomes beyond programme area of focus); sustainability (sustained results over time); spreadability (diffusion or spread of programme influence beyond individual programme participants); and scalability (programme expansion or replication; appendix p 15). Among these high-quality programmes, we used thematic analysis to identify broad similarities in programme implementation.

### Role of the funding source

The funder of the study had no role in study design, data collection, data analysis, data interpretation, or writing of the report. The corresponding author had full access to all the data in the study and had final responsibility for the decision to submit for publication.

## Results

**Figure 1:**
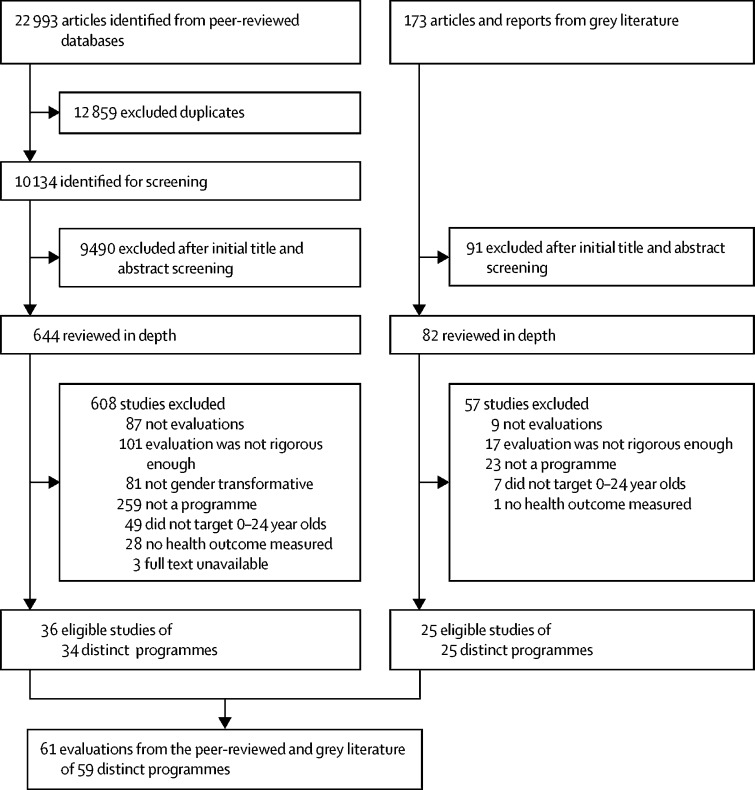
Study selection

Our search of peer-reviewed literature identified 22 993 articles, from which further screening identified 36 evaluations of 34 distinct programmes meeting our inclusion criteria. Our internet search retrieved 173 potential evaluations, among which we identified 25 distinct programme evaluations that met the same inclusion criteria used for the peer-reviewed articles. Altogether, we reviewed 61 evaluations of 59 programmes (figure 1, appendix pp 44–49).

**Figure 2:**
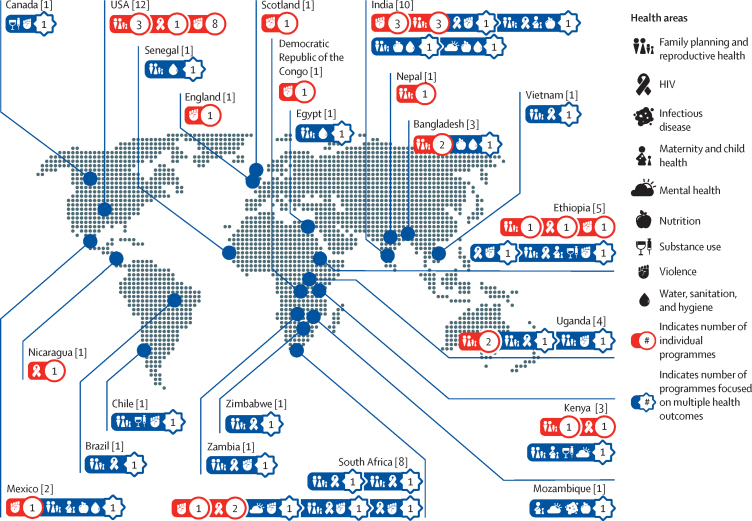
Geographical distribution of programmes and the health areas they addressed

33 (54%) of the 61 evaluations that met our screening criteria used exclusively quantitative approaches, 27 (44%) used mixed methods, and one used only qualitative methods (appendix p 16–43). 28 (47%) of the quantitative and mixed-method approaches were RCTs, and 31 (52%) were quasi-experimental. 33 (54%) of the evaluations included a programme sample of at least 1000 participants. 25 (42%) of 59 programmes were in sub-Saharan Africa, 13 (22%) were in south Asia, and 13 (22%) in North America (figure 2), and the majority were implemented in either urban-only areas (23 [39%]) or included both urban and rural populations (15 [25%]).

55 (90%) of the 61 evaluations measured outcomes related to family planning or sexual and reproductive health (29 [48%]), physical, sexual, or emotional violence (26 [43%]), and HIV (18 [30%]). Programme evaluations also measured outcomes related to nutrition (seven [12%] of 61); maternal and child health (five [8%]); water, sanitation, and hygiene (five [8%]); mental health (four [7%]); substance use (four [7%]); and infectious disease (one [2%]). 25 (41%) of the programme evaluations measured two or more areas of health at once; 14 (56%) of these 25 evaluations were in sub-Saharan Africa (figure 2).

50 (82%) of 61 evaluations measured outcomes among children and adolescents aged 10–14 years (44 [72%]) or 15–19 years (48 [79%]) as either targeted participants or beneficiaries of the programme. 33 (54%) of the 61 studies included community members, caretakers, parents, or teachers to enhance programme outcomes, and five (8%) focused on outcomes among children below the age of 10 years. Six (10%) evaluations were of programmes that targeted young adults aged 20–24 years and aimed to improve sexual and reproductive health outcomes, including HIV.

More than half of the programmes (33 [56%] of 59) engaged men or boys through activities to improve interpersonal skills, shift notions of masculinity, decrease violence, and redefine roles and responsibilities within the household. 11 (33%) of these 33 programmes focused on males as the primary beneficiary population and were designed to improve health among males specifically; the other 23 (67%) programmes implemented activities to primarily benefit females.

A few programmes addressed intersectionality across ethnicity, socioeconomic status, or other social categories. Eight (14%) of 59 programmes focused primarily on gender inequality among one or more of their country's minority ethnic populations and 12 (20%) worked with subpopulations living in relative poverty. Other targeted social groups included those affected by conflict, married and unmarried girls, married men, newly married couples, orphans, out-of-school girls and boys, and university students. No programme exclusively targeted gender minorities or LGBTQ populations.

**Figure 3:**
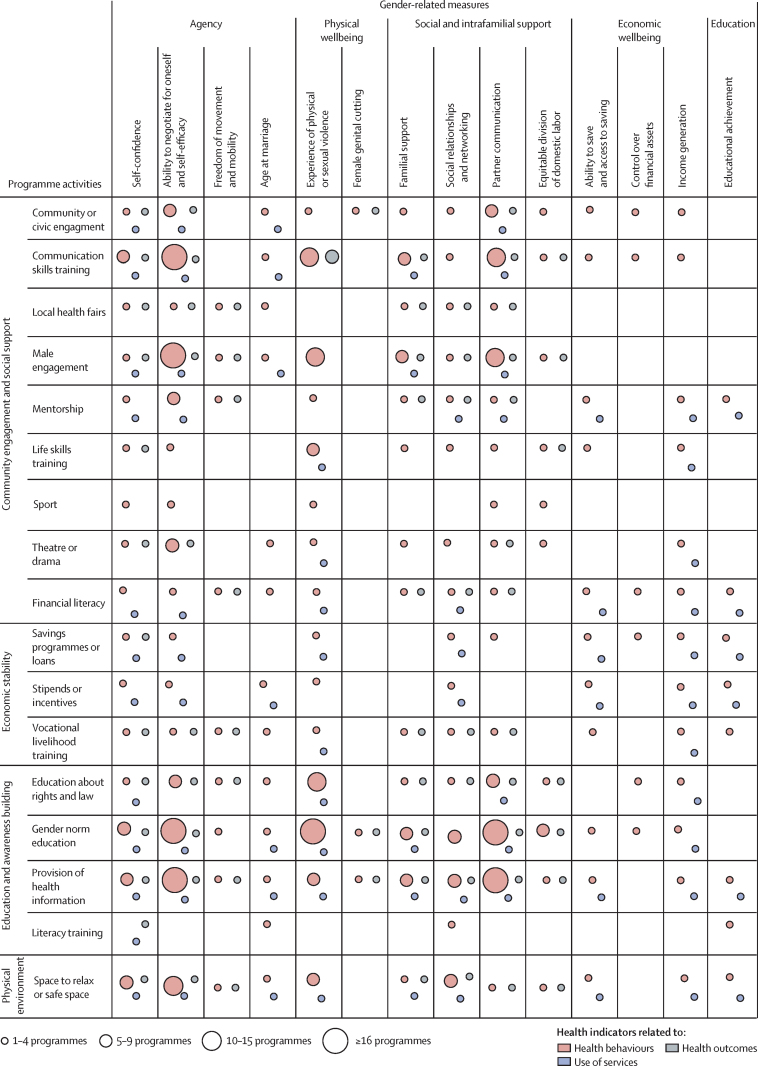
Landscape analysis of programmes targeting health-related service utilisation, behaviours, and outcomes by programme activities and the gender-related indicators they measured

Figure 3 describes the landscape of programme activities (grouped by the Social Determinants of Health Framework),^11^ as well as the gender-related and health-related indices that were measured. Most (57 [97%] of 59) of the programmes implemented were interactive educational or awareness-building activities fostering critical consciousness of existing norms and inequalities, creating space for community engagement and debate, and stimulating discussion of how gender norms might advantage or limit opportunities for an individual. The programme activities covered several different topics: local, restrictive gender norms (48 [81%] of 59); health education (44 [75%]); laws and policies and the rights and entitlements of the individual (17 [29%]), and literacy training (four [7%]).

Activities also often focused on engaging communities or building social support systems (53 [90%] of 59). For example, programmes included community events such as theatre and drama productions (11 [19%]) and local health fairs (one [2%]); and facilitated social integration through life skills training (17 [29%]), community and civic engagement (11 [19%]), mentorship or peer support (ten [17%]), and sports (five [9%]). Activities such as role-playing allowed participants to build communication skills and rethink norms related to the right to express opinions and negotiate choices (33 [56%]).

14 programmes (24%), all in sub-Saharan Africa (nine) and south Asia (five), addressed the problems that women and girls often face in not having access to and control over resources. These programmes aimed to enhance the economic stability of these women and girls by building their financial literacy and providing access to savings programmes, loans, stipends, incentives, and vocational livelihood training. 28 programmes (47%) in sub-Saharan Africa (14), south Asia (11), and the Middle East (three) provided safe spaces for participants to explore sensitive topics or to safely socialise and build social networks. In North America, three programmes implementing these activities focused on violence prevention; in Latin America, one programme provided a safe space for boys to discuss issues like violence, relationships, and family. Two programmes explicitly mentioned involving religious leaders as allies, and all programmes implemented activities within or through secular spaces.

Rather than focusing on systemic inequity, programmes most frequently implemented activities meant to improve the power and influence that beneficiaries have over their own lives. More than half of the evaluations (34 [56%] of 61) assessed changes in the agency of participants, measuring indicators related to self-confidence, self-efficacy, freedom of movement, and age at marriage.^17^ Five evaluations (8%) of 61 measured educational attainment and nine (15%) measured changes in the economic wellbeing of participants, both of which are strong predictors of gender equality and health at the aggregate level. Changes in gender-related attitudes and beliefs, which are often conflated with norm change,^22^ were measured in 36 (59%) of the 61 evaluations, and nine (15%) measured changes in gender-related knowledge. Approximately half of the evaluations (30 [49%]) measured variables related to changes in social and intrafamilial support (appendix p 16–43).

Almost three-quarters (45 [74%]) of the evaluations showed significant improvements in indicators of health and measures related to gender inequality, of which 32 (71%) were evaluations of programmes that targeted people aged 10–19 years as their primary beneficiary population. 31 (69%) of these 45 studies showed changes in knowledge about health topics such as sexually transmitted infections, contraception, and body satisfaction; 38 (84%) showed changes in health behaviours, such as substance abuse, breastfeeding practices, and exercise; and four (9%) showed significant improvements in the use of health services. Only 13 (21%), however, actually measured health outcomes—rather than knowledge, attitudes, or behaviour related to health—and found significant decreases in the incidence of unwanted or unintended pregnancy (five [8%]), HIV (3 [5%]), female genital mutilation or cutting (FGMC; one [2%]), stunting (two [3%]), child morbidity (one [2%]), and violence (five [8%]) or anxiety (one [2%]).

Regardless of their indicators of health, these 45 evaluations were of programmes that relied on activities mainly targeting mechanisms that enforce gender norms and are related to agency (27 [60%]), social and intrafamilial support (23 [51%]), and attitudes towards restrictive gender norms (28 [62%]), including gender norms related to violence, FGMC, and early marriage. Almost two-thirds of these 45 programmes were implemented for 12–24 months (11 [24%]) or more (17 [38%]). Programmes that were implemented for shorter periods, yet still produced significant outcomes, mainly measured changes in health knowledge or behaviours related to condom use, risky sex, or perpetration of violence.

These 45 programmes were primarily implemented in the community (27 [60%]), as part of a school curriculum (13 [29%]), or as part of after-school programmes (14 [31%]). One (2%) incorporated activities in a prison, and one (2%) was implemented within a health-care setting. Regardless of their setting, these programmes used diverse methods to disseminate information and challenge the long-held beliefs of participants, including role play, dialogue and discussion, peer-to-peer activities, circulation of print materials, audiovisual media (ie, radio and television), and digital media. Although the diversity of the programmes made it impossible to compare effective combinations of approaches, 20 (44%) implemented three or more of these activities to accomplish their goals.

Among the 45 programmes that brought about significant changes in their measured outcomes related to health and gender, 14 (31%) implemented activities that engaged girls and boys in the same activity at the same time. This approach enabled participants to engage with members of another sex in a safe, structured setting to explore, discuss, and reframe gender roles, assumptions, and decision making. However, in settings with restrictive gender norms and little informal contact between women and men, introducing topics in segregated groups is important.^23^, ^24^ In such situations, female and male participants met separately to gain confidence and address normally stigmatised topics, then came together to facilitate shared understanding of the issues, develop alliances, and collaboratively strategise about how to create change.

Only ten (17%) of all 59 programmes showed evidence of, or potential for, broad change in gender norms (appendix p 15; table). These high-quality programmes reflected four mutually reinforcing factors: (1) through multisectoral action, the programmes worked with sectors beyond health to create change; (2) the programmes included the participation of multiple stakeholders at different levels of the Social Ecological Model;^35^ (3) the programmes implemented diversified programming, combining activities that reinforce one another and address issues from multiple perspectives; and (4) through social participation and empowerment, the programmes fostered critical awareness and participation among affected community members, encouraging them to become active agents in shaping their own health.

**Table:** High-quality gender-transformative programmes

	**Programme description**	**Evaluation design**	**Health-related evidence**	**Gender-related evidence**
Sexuality Education Initiative; Los Angeles, CA, USA; Constantine et al (2015)^25^	The Sexuality Education Initiative aimed to improve the sexual and reproductive health of high-school students by engaging both students and their parents in a 12-week classroom-based curriculum; students served as peer advocates and had access to sexual health services on school grounds; parents received educational workshops and tailored materials on sexual and reproductive health	Design: cluster RCT with ten high schools in Los Angeles; data collection: 1750 students (intervention group 934; control group 816) surveyed at pre-intervention, immediately after the intervention, and at 1-year follow-up	Significant effect: increased knowledge about sexual health and sexual health services (p<0·001); non-significant effects: increased communication with sexual partners and increased intentions to use condoms	Significant effects: more positive attitudes about sexual relationship rights (adjusted standardised mean difference 0·42); greater self-efficacy to manage risky situations (adjusted standardised mean difference 0·37)
The PRACHAR Project; Bihar, India; Daniel and Nanda (2012)^26^	PRACHAR sought to increase the age of marriage for girls, delay first birth after marriage, and ensure birth spacing by improving awareness, knowledge, and understanding of reproductive health issues among adolescents aged 15–19 years, their guardians, and influential community members; information was communicated through various channels, including educational workshops, parties, home visits, wall paintings, and pamphlets; community mobilisation activities were used to foster support for programme concepts	Design: cluster RCT with 20 villages; data collection: randomly selected adolescents (intervention group 613; control group 612) surveyed 5 years after the intervention	Significant effects: increased odds (aOR 4·95) of contraception use after marriage and before first birth among women (p<0·001), increased odds (aOR 3·58) of contraception use after marriage among married men (p<0·001), age at first birth for women 1·5 years higher (p<0·001); decreased odds (RR 0·61) of having had a birth at 5-year follow-up (p<0·01)	Significant effects: decreased odds (0·56) of women being married at 5-year follow-up (p<0·001); decreased odds (0·74) of men being married at 5-year follow-up (p<0·05); increased educational attainment among women (p<0·05) and men (p<0·001); non-significant effects: increased odds that women (aOR 6·10) and men expressed to their parents the age at which they wanted to marry (aOR 5·30)*
TOSTAN; Kolda Region, Senegal; Diop et al (2004)^27^	The goal of TOSTAN was to improve the health of women and encourage the abandonment of FGMC and forced child marriage through education, empowerment, and social mobilisation; programme activities required participation from stakeholders across multiple levels of society and included a four module education programme, discussions with community leaders, capacity building with local non-governmental organisations, training for local community members, and a public declaration renouncing FGMC	Design: mixed-methods quasi-experimental trial with 40 villages and three comparison groups (individuals directly exposed, indirectly exposed, and not exposed to the programme); data collection: 2397 individuals (directly exposed 967; indirectly exposed 692; not exposed 738) surveyed pre-intervention, immediately after the intervention, and 2 years after the intervention	Significant effects: increased use of antenatal services (p<0·001) and increased proportion of girls aged 0–10 years who had not been cut (p<0·05) in individuals who had been directly or indirectly exposed to the intervention compared with individuals who had not been exposed	Significant effects: awareness of human rights increased by 83% for women and 51% for men (p<0·001) directly exposed to the intervention; decreased proportion of men intending to have their daughters cut (p<0·001) in all three groups after the intervention compared with baseline, the degree of change was greatest in men directly exposed to the intervention; decreased proportion of women reporting experience of violence in the past 12 months (p<0·001) in all groups after the intervention compared with baseline, the degree of change was greatest in women directly or indirectly exposed to the intervention
Shaping the Health of Adolescents in Zimbabwe Project (SHAZ!); Chitungwiza, Zimbabwe; Dunbar et al (2014)^28^	SHAZ! was an HIV prevention programme for out-of-school girls aged 16–19 years who had lost at least one parent; the programme addressed structural barriers to prevention by providing participants with sexual and reproductive health services, life skills and home-based care training, livelihoods training, and guidance counselling or adult mentorship	Design: RCT; data collection: 315 girls (intervention group 158; control group 157) surveyed pre-intervention and every 6 months after for 24 months	Significant effects: lower food insecurity in intervention group (OR 0·83) *vs* in control group (OR 0·68, p=0·02); non-significant effects: reduced risk of transactional sex (OR 0·64, p=0·25), higher likelihood of using a condom with their current partner (OR 1·79, p=0·25), fewer unintended pregnancies (HR 0·61, p=0·061), decreased HIV incidence (HR 0·94, p=0·913)	Significant effects: increased odds of individuals having their own income (p=0·02); non-significant effects: reduced experience of violence over time (p=0·06)
Development Initiative Supporting Healthy Adolescents (DISHA); Bihar and Jharkhand, India; Kanesathasan et al (2008)^29^	DISHA aimed to improve sexual and reproductive health outcomes for young people aged 10–24 years by addressing social and economic influences; the programme provided health information and services directly to young people and training to sexual and reproductive health service providers; a media campaign was implemented to influence community awareness	Design: mixed-methods, quasi-experimental study with 176 villages; data collection: 4645 young people aged 14–24 years and 1601 women and men older than 30 years, surveyed pre-intervention and again 3 years later; 36 focus groups	Significant effects: increased knowledge of how to access the pill among married young women (p<0·05); increased knowledge of condoms among young people (p<0·05); decreased number of young women who disapproved of contraceptive use among married couples (p<0·0001); increased number of young people who believed that contraceptives should be available to young married couples (p<0·05); all in the intervention groups compared with the control group	Significant effects: increased proportion of young women (63%) and young men (72%) who knew the legal age of marriage for girls (p<0·05); increased odds that unmarried young people were able to talk with their elders about marriage timing (p<0·05); increased reported ability of young women to access sexual and reproductive health services unaccompanied (p<0·05); non-significant effects: increased ability of married girls to speak with spouse about contraception; all in the intervention groups compared with the control group
Primary School Action for Better Health (PSABH); Nyanza Province, Kenya; Maticka-Tyndale et al (2007)^30^	PSABH aimed to reduce the risk of HIV infection among school students aged 11–16 years through delaying sexual debut, decreasing sexual activity, and increasing condom use; through a train-the-trainer model, teachers who attended training on the sexuality and HIV prevention programme trained their colleagues to integrate sexuality and HIV into the existing school curriculum	Design: mixed-methods quasi-experimental study with 80 schools; data collection: 3940 students (intervention group 1964; control group 1976) surveyed pre-intervention and 18 months after the intervention; 16 focus group discussions facilitated with 320 students from 24 schools; 48 qualitative interviews with teachers from 24 schools	Significant effects: students three times more likely to report high levels of exposure to HIV and AIDS education (p<0·001), increased HIV and AIDS knowledge among boys who were virgins before programme implementation (p<0·05), increased condom use at last intercourse among boys (p<0·05)	Significant effects: increased self-efficacy among girls sexually active before programme implementation, as shown by greater likelihood of reporting they could say no to sex (p<0·001) and could have a boyfriend and not have sex (p<0·001); qualitative findings: boys and girls with and without history of sexual intercourse responded differently for establishing alternative scripts related to negotiation and refusal of unwanted sex; girls describe strategies to refuse sex and avoid or leave relationships; boys showed more programme-related gains in condom self-efficacy than girls; girls who had not had sexual intercourse showed higher self-efficacy for future condom negotiation than girls who had had sex
Program H; Rio de Janeiro, Brazil; Pulerwitz et al (2006)^31^	The goal of Program H was to reduce the risk of HIV and sexually transmitted infections among low-income, in-school and out-of-school boys and young men aged 14–25 years by challenging gender norms around masculinity, reflecting on the consequences of inequitable gender norms, and promoting gender-equitable behaviours; this was accomplished through gender-equitable messaging in 6-month group education for boys, a community-wide lifestyle social marketing campaign, and peer promoters	Design: mixed-methods quasi-experimental study with two sites and three study groups (group education only, combined activities, and no activities); data collection: Gender-Equitable Men Scale surveys among 780 boys (258 group education only; 250 combined activities; 272 control group) at pre-implementation and at 6 and 12 months post-implementation; 18 in-depth qualitative interviews with participants and their female sexual partners	Significant effects: increased condom use at last sex with a primary partner at 6-month follow-up among young men in both intervention groups and maintained at 12 months in the combined activities intervention group (p<0·05), decreased reported sexually transmitted infection symptoms in the combined activities intervention group at follow up at 6 months and maintained at 12 months (p<0·05); non-significant effects: condom use increased with casual partners in all three study groups, reported sexually transmitted infection symptoms decreased compared with the previous 3 months in the group education-only intervention group and the control group	Significant effects: decrease in the proportion of respondents who supported inequitable gender norms maintained to 12-month follow-up at both intervention sites (p<0·05) compared with the control; non-significant effects: increased partner communication about HIV and condoms in both intervention groups compared with the control; qualitative findings: group education sessions served as safe spaces for young men to discuss issues not typically spoken about (eg, community violence, relationships, and family life)
Strengthening Household Ability to Respond to Development Opportunities Project (SHOUHARDO); North Char, Mid Char, Haor, and Coast, Bangladesh; Smith et al (2011)^32^	The goal of SHOUHARDO was to reduce child malnutrition, poverty, and food insecurity in the poorest households with children aged 6–24 months, by addressing the underlying structural causes of poverty; through a multipronged, rights-based, livelihoods approach, the programme implemented interconnected activities to improve health, sanitation, food insecurity, education for women and girls, women's empowerment, income-generating activities, and access to financial resources	Design: mixed-methods quasi-experimental study using difference-in-difference propensity score matching; data collection: surveys completed by 3200 households with children 6–24 months old pre-intervention and 3 years later by 3200 households with children 48–59 months old in the same villages as baseline and 3356 new households with children 6–24 months old; collection of secondary data from national survey	Significant effects: increased food security (p<0·001); increased care practices for children 6–24 months, including fully immunising children before 1 year of age; vitamin A supplementation, oral rehydration therapy during diarrhoea (p<0·001 for all), and breastfeeding (p<0·05); increased care practices for mothers, including three antenatal visits, iron, folic acid, and vitamin A supplementation, food intake during pregnancy, and daytime rest (p<0·001 for all); increased access to safe water and sanitary latrine (p<0·001) in households in the SHOUHARDO project area compared with national data on rural households with young children; non-significant effects: 15·7% decrease in stunting prevalence and increase in stunting among children aged 6–18 months and 48–60 months in households in the SHOUHARDO project area compared with national data on rural households with young children	Significant effects: the women's empowerment activities had an independent effect on stunting (p<0·001); increase in women taking more daytime rest than usual during last pregnancy (% difference 82·2, p<0·001); synergistic effects between maternal, neonate, and child health activities on stunting were women's empowerment activities (p<0·001), sanitation activities (p<0·05), and poverty alleviation activities (p<0·05); all in households in the SHOUHARDO project area compared with national data on rural households with young children; triangulated findings: increased equitable access to land; increased number of income-generating opportunities, savings mechanisms, and access to credit; improved life skills of men and women and higher employability of girls; increased gender equity at family and community level in households in the SHOUHARDO project area compared with national data on rural households with young children
Somos Diferentes, Somos Iguales (SDSI); Estelí, Juigalpa, and León, Nicaragua; Solórzano et al (2008)^33^	SDSI aimed to prevent HIV infection in young people aged 10–25 years by addressing social and cultural barriers to prevention; the programme included a national television series (Sexto Sentido), a nightly call-in radio show for young people, a media campaign, information, education, and communications materials, analytical and leadership skills training related to sexual and reproductive health and rights issues for youth leaders, and alliance building between young people and adults in various sectors	Design: mixed-methods quasi-experimental longitudinal study in three cities; data collection: 3099 young people aged 13–24 years surveyed at pre-intervention and at 1 year and 2 years post-intervention; 45 in-depth interviews with participants and non-participants (39 with key stakeholders); ten group interviews with representatives from local organisations; 20 focus group discussions with participants and non-participants	Significant effects: exposure to SDSI increased use of services related to HIV and intimate partner violence (OR 1·48, 95% CI 1·2–1·9); increased likelihood of consistent condom use with casual partners (OR 1·42, 95% CI 1·1–1·9) among participants with greater exposure to the intervention; increased interpersonal communication about domestic violence, HIV, homosexuality, condom use, and the rights of young people (OR 1·6, 95% CI 1·5–1·8) among participants with greater exposure to the intervention compared with those with less exposure to SDSI; non-significant effects: condom use with steady partners increased among all groups	Significant effects: higher scores on gender-equitable attitudes associated with greater exposure (ie, watched at least 2 seasons of the television series) to SDSI (p<0·001); less stigmatising attitudes towards homosexuality and people with HIV associated with greater exposure to SDSI (p<0·001); higher perceived self-efficacy to negotiate condom use in those with greater exposure to the intervention compared with those with less exposure (p=0·033); increased partner communication about HIV prevention in those with greater exposure to the intervention compared with those with less exposure (OR 1·43, 95% CI 1·2–1·7)
Fourth R: Skills for Youth Relationships; ON, Canada; Wolfe et al (2009)^34^	Fourth R was a physical dating violence reduction programme for high school students aged 14–15 years; in addition to a 21-lesson educational curriculum on healthy relationships, the programme engaged students' parents, provided teachers with trainings on dating violence and healthy relationships, and created student-led safe-school committees	Design: cluster RCT with ten public schools; data collection: online surveys completed by 1722 students (intervention group 968; control group 754) pre-intervention and 2·5 years after the intervention	Significant effects: decreased likelihood that boys would perpetrate physical dating violence (aOR 0·36, p=0·05); decreased experience of physical dating violence (aOR 0·41, p=0·05); increased condom use among sexually active boys (OR 1·70; p=0·05); non-significant effects: decreased engagement in peer physical violence (eg, arguments, threats of violence, hurting another with the intention to humiliate, slapping); increased condom use among all students; decreased problem substance use	Observational findings: improved negotiation skills and more equitable decision making within relationships

These results are those that authors highlight in their evaluation reports. If the programme was attempting to decrease violence, then results related to violence were included as health-related outcomes. If the programme was attempting to decrease violence as a mechanism for influencing other outcomes (eg, HIV), then changes in violence were included as gender-related evidence. aOR=adjusted odds ratio. FGMC=female genital mutilation or cutting. HR=hazard ratio. OR=odds ratio. RCT=randomised controlled trial. RR=relative risk.

* p value not reported.

## Discussion

This systematic review assessed rigorously evaluated programmes that sought to transform gender relations to promote equality and achieve health and wellbeing among people aged 0–24 years, living in any part of the world. These gender-transformative programmes exemplify the Sustainable Development Goal principle of investing in areas that spark or catalyse diffusion of change.^14^ Approximately three-quarters of the 61 evaluations (45 [74%]) reported significant changes in variables related to both gender and health, and ten (16%) showed outcomes that are associated with broader gender norm change. Thematic analysis of the evaluated programmes revealed a potential model for accelerating achievement of the Sustainable Development Goals through programmatic approaches that improve health-related outcomes and seek to achieve gender equality and broader transformation of restrictive gender norms. Four such programmatic approaches were identified: (1) using multisectoral action; (2) incorporating multilevel, multistakeholder involvement; (3) implementing diversified programming; and (4) fostering social participation and empowerment.

**Figure 4:**
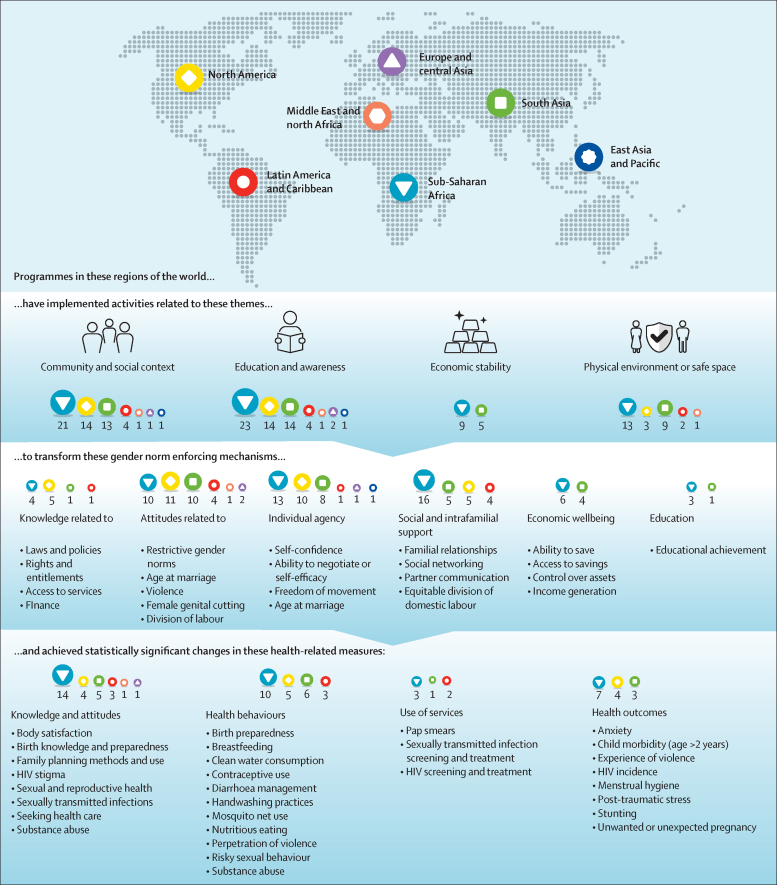
Programme operationalisation by region Numbers indicate which regions implemented specific activities.

Although this systematic review shows the potential contributions that gender-transformative programming can have on gender equality and health, our analysis found several gaps in current programme design, implementation, and evaluation. Figure 4 shows our synthesis of theoretical pathways between programme activities, the gender-norm-enforcing mechanisms they targeted, and the health-related variables for which there was a significant change. Every region had at least one programme that implemented activities that involved raising critical awareness around restrictive gender norms, engaging the community, building social support systems, or a combination of these activities. Despite often framing decreasing gender inequality and improving gender norms as structural or systemic challenges, these activities aimed to improve health-related outcomes by primarily shifting individual and interpersonal indicators related to participants' agency and taken-for-granted, gender-related attitudes and behaviours. Although some programmes succeeded in achieving significant improvements in their gender and health measures, this approach of targeting individual and interpersonal attitudes did not necessarily lead to systemic change in gender equality or norms. In some programmes, this approach might even have led to unmeasured backlash, with participants facing community sanctions when they stood up against prevailing social pressures.^36^

The same limitation applied to downstream health outcomes, which are often driven by external forces as well as internal decisions and power. For example, evaluations of programmes that attempted to improve attitudes to violence (21 [34%] of 61) found significant improvement 81% of the time, and evaluations of programmes that attempted to improve behaviours related to violence (18 [30%]) found significant improvement 72% of the time. However, only five (38%) of the 13 programmes that measured participants' experience of violence showed significant improvement. Thus, societal and structural elements of restrictive gender norms often overpower efforts to improve a single aspect of individual or even interpersonal power, making health outcomes difficult to change without a more holistic, systems approach.^37^

The programmes in our review were also potentially limited by their narrow health focus. Most programme evaluations (55 [90%] of 61) were designed to measure only outcomes related to HIV, sexual and reproductive health, or violence, which is problematic given the growing evidence that gender norms influence many different health areas.^7^ As the global burden of disease has shifted among young people, the underlying gender norms related to a broad range of health outcomes, including mental health, infectious disease, substance use, injury, and chronic diseases, must be addressed.

Programmes were also limited by their poor inclusivity of activities and beneficiary participation. Programmes succeeded at producing significant changes in indicators related to gender or health when the implemented activities facilitated joint reflection and allyship among girls and boys or women and men. Yet, even the high-quality programmes identified in our review often emphasised the empowerment of girls and women to the comparative neglect of masculinity and restrictive gender norms that affect sexual orientation and gender identity and expression. Furthermore, very few gender-transformative programmes were working to improve health among gender-diverse people.

Our research also highlights the need to understand gender in the context of intersectionality—the overlapping effects of different aspects of status and identity—which can magnify the influence of gender inequalities and restrictive gender norms on health. Although programmes in our review often focused on disadvantaged subpopulations, fewer than 10% explored the specific contributions of race and ethnicity, religion, or geographic location. Furthermore, only one programme primarily targeted a population of children younger than 10 years,^38^ an important gap given the potential advantages of working with younger children and their influencers.

In addition to design and implementation gaps in the programmes themselves, we identified opportunities for improving the evaluation of such programmes. Given that they appear to work upstream from several positive outcomes, programmes that seek to transform norms have the potential to improve health and wellbeing across many areas of health and for long periods across the life course. Yet, little is known about the full effect of these programmes, because the majority of the 61 evaluations in our review measured outcomes related to one area of health (36 [59%]) and evaluated outcomes during implementation or within 6 months of programme completion (36 [59%]). Only four (7%)—all of which produced significant, sustainable change—evaluated the outcomes more than 3 years after programme completion. Furthermore, very few evaluations triangulated their measures by collecting data on the same outcome from multiple sources. Given the social nature of gender norms, understanding and measuring effects beyond the individual is important. As programmes increasingly address gender inequality and restrictive gender norms to improve health and other aspects of wellbeing, innovative and comprehensive ways of measuring norm change are needed.

We could have missed programmes that might be considered gender transformative but did not indicate as such in the title or abstract or their evaluation. Moreover, we might have missed strong programmes that improve health through gender-transformative work but did not measure health-related outcomes. For example, programmes that aimed to end child marriage were excluded unless they measured an associated health outcome. Our systematic review was also restricted to methods suitable for examining causal effect. The hierarchy of evidence developed for clinical interventions might not be ideally suited for complex social interventions because it does not fully account for complexity and non-linear causality. We might thus have excluded effective programmes that were small or did not otherwise meet our evaluation criteria. Moreover, the evidence available might be biased because financial donors have preferences in the health areas, geographies, interventions, and research methods that they fund. Additionally, we were able to analyse only what was measured and reported in the evaluation publications. The disparate indicators and measurement and analysis techniques used in the different studies made it impossible to compare magnitude of change or generate summative statistics.

Future research could build on this study by focusing more explicitly on evaluations that use rigorous qualitative methods to further unpack specific mechanisms of change. An important programmatic and research challenge is to conceptualise and address gender inequalities and restrictive gender norms that affect gender minorities or LGBTQ populations. What are the attitudes and behaviours of the majority population that create the conditions of stigma and exclusion for people who depart from the norm? How can gender-transformative programmes address such issues?

An initial aspiration of this study was to assess the contributions of gender-transformative programmes to reducing the causes of morbidity and mortality that contribute most heavily to the global burden of disease. Although we found a continued focus on HIV, sexual and reproductive health, and violence, as the global burden of disease shifts so does the opportunity to implement gender-transformative programmes in other areas of health and development. Indeed, programmes that decrease gender inequality and transform restrictive gender norms are arguably key to achieving the Sustainable Development Goals.^2^, ^14^
