# Supplementary material for: Characteristics of successful programmes targeting gender inequality and restrictive gender norms for the health and wellbeing of children, adolescents, and young adults: a systematic review
Source: Lancet Glob Health. 2019 Dec 23;8(2):e225–36. doi: 10.1016/S2214-109X(19)30495-4 (PMC7025324; doi:10.1016/S2214-109X(19)30495-4)
Supplement: Supplementary appendix [file mmc1.pdf]

THE LANCET
Global Health

Supplementary appendix

This appendix formed part of the original submission and has been peer reviewed. We post it as supplied by the authors.

Supplement to: Levy JK, Darmstadt GL, Ashby C, et al. Characteristics of successful programmes targeting gender inequality and restrictive gender norms for the health and wellbeing of children, adolescents, and young adults: a systematic review. *Lancet Glob Health* 2019; published online Dec 23. http://dx.doi.org/10.1016/S2214-109X(19)30495-4.

Appendix: PRISMA Checklist

Section/topic	#	Checklist item	Page #
**TITLE**			
Title	1	Identify the report as a systematic review, meta-analysis, or both.	1
**ABSTRACT**			
Structured summary	2	Provide a structured summary including, as applicable: background; objectives; data sources; study eligibility criteria, participants, and interventions; study appraisal and synthesis methods; results; limitations; conclusions and implications of key findings; systematic review registration number.	1
**INTRODUCTION**			
Rationale	3	Describe the rationale for the review in the context of what is already known.	2
Objectives	4	Provide an explicit statement of questions being addressed with reference to participants, interventions, comparisons, outcomes, and study design (PICOS).	2, 3, App p.3
**METHODS**			
Protocol and registration	5	Indicate if a review protocol exists, if and where it can be accessed (e.g., Web address), and, if available, provide registration information including registration number.	3, 12
Eligibility criteria	6	Specify study characteristics (e.g., PICOS, length of follow-up) and report characteristics (e.g., years considered, language, publication status) used as criteria for eligibility, giving rationale.	2, 3, App p.3-4
Information sources	7	Describe all information sources (e.g., databases with dates of coverage, contact with study authors to identify additional studies) in the search and date last searched.	3, App p.3
Search	8	Present full electronic search strategy for at least one database, including any limits used, such that it could be repeated.	App p.3
Study selection	9	State the process for selecting studies (i.e., screening, eligibility, included in systematic review, and, if applicable, included in the meta-analysis).	2, 3 App p.3-4
Data collection process	10	Describe method of data extraction from reports (e.g., piloted forms, independently, in duplicate) and any processes for obtaining and confirming data from investigators.	3, App p.8-14
Data items	11	List and define all variables for which data were sought (e.g., PICOS, funding sources) and any assumptions and simplifications made.	App p. 3-14
Risk of bias in individual studies	12	Describe methods used for assessing risk of bias of individual studies (including specification of whether this was done at the study or outcome level), and how this information is to be used in any data synthesis.	3

Summary measures	13	State the principal summary measures (e.g., risk ratio, difference in means).	2, 3
Synthesis of results	14	Describe the methods of handling data and combining results of studies, if done, including measures of consistency (e.g., I^2) for each meta-analysis.	NA
Risk of bias across studies	15	Specify any assessment of risk of bias that may affect the cumulative evidence (e.g., publication bias, selective reporting within studies).	11, Pan 1
Additional analyses	16	Describe methods of additional analyses (e.g., sensitivity or subgroup analyses, meta-regression), if done, indicating which were pre-specified.	NA
**RESULTS**			
Study selection	17	Give numbers of studies screened, assessed for eligibility, and included in the review, with reasons for exclusions at each stage, ideally with a flow diagram.	Fig 1
Study characteristics	18	For each study, present characteristics for which data were extracted (e.g., study size, PICOS, follow-up period) and provide the citations.	Tab 1
Risk of bias within studies	19	Present data on risk of bias of each study and, if available, any outcome level assessment (see item 12).	3
Results of individual studies	20	For all outcomes considered (benefits or harms), present, for each study: (a) simple summary data for each intervention group (b) effect estimates and confidence intervals, ideally with a forest plot.	NA
Synthesis of results	21	Present results of each meta-analysis done, including confidence intervals and measures of consistency.	3-10
Risk of bias across studies	22	Present results of any assessment of risk of bias across studies (see Item 15).	11, Pan 1
Additional analysis	23	Give results of additional analyses, if done (e.g., sensitivity or subgroup analyses, meta-regression [see Item 16]).	NA
**DISCUSSION**			
Summary of evidence	24	Summarize the main findings including the strength of evidence for each main outcome; consider their relevance to key groups (e.g., healthcare providers, users, and policy makers).	10-11, Fig. 4
Limitations	25	Discuss limitations at study and outcome level (e.g., risk of bias), and at review-level (e.g., incomplete retrieval of identified research, reporting bias).	11
Conclusions	26	Provide a general interpretation of the results in the context of other evidence, and implications for future research.	11
**FUNDING**			
Funding	27	Describe sources of funding for the systematic review and other support (e.g., supply of data); role of funders for the systematic review.	3, 12

Appendix:

PICO criteria for inclusion

Domain	Criteria
Population	• Targets or beneficiaries of the programme who are:<br>o Children, adolescents, or youth aged 0-24<br>o Any sex or gender<br>o Anywhere in the world
Intervention	• Non-policy related health programme or research intervention that:<br>o Has been rigorously evaluated; AND<br>o Meets at least one of the criteria of the Interagency Gender Working Group's (IGWG) definition of 'gender-transformative' programming: 'programmes that seek to transform gender relations to promote equality and achieve program objectives […] by: 1) fostering critical examination of inequalities and gender roles, norms, and dynamics; 2) recognizing and strengthening positive norms that support equality and an enabling environment; 3) promoting the relative position of women, girls, and marginalized groups; and 4) transforming the underlying social structures, policies, and broadly held social norms that perpetuate gender inequalities; AND<br>o Is health related (e.g. focused on improving health knowledge, attitudes, behaviours, and/or outcomes)
Comparison	• Health- and gender-related measures among those not exposed to the programme
Outcome	• Evaluations must measure changes in – at a minimum – any health-related outcome, and – at best – both health- and gender- related outcomes. Specifically:<br>o Health measures may include short, intermediate, or long-term indicators within any area of health, such as: chronic disease; infectious disease; maternal and child health; mental health; nutrition; physical activity; sexual and reproductive health; substance abuse; unintentional injury; violence; or water, sanitation and hygiene.<br>o Gender-related measures may include those pertaining to: knowledge, attitudes, beliefs and/or behaviours linked to the restrictive gender norms in participants' settings; measures of individual autonomy; interpersonal social support systems and power dynamics; and social structures, laws, policies, or systems that influence gender inequality and/or restrictive gender norms.

Questions to screen for inclusion of programme evaluations

Exclusionary questions	YES	NO	Unclear
Initial exclusionary criteria (titles and abstracts in databases)			
1. Was the article published after 2000?			
IF NO, THEN EXCLUDE			
2. Is the article in English, French, Spanish, or Portuguese?			
IF NO, THEN EXCLUDE			
3. Is the study a peer-reviewed journal article? Is it a grey literature publication, such as a working paper, technical report, government document, or white paper?			
IF NO, THEN EXCLUDE			
4. Is the study a review of interventions (ie, another systematic review or a meta-analysis)?			
IF YES, THEN EXCLUDE (but use to identify interventions)			
5. Are the programme participants below the age of 24? (ie, either as the target or beneficiary population)?			
IF NO, THEN EXCLUDE			
6. Does the article describe a non-policy health intervention (ie, a research or programmatic intervention focused on changing a health-related behaviour or outcome)?			
IF NO, THEN EXCLUDE			
7. Is the health intervention gender aware (ie, does it explicitly recognise local gender power imbalances, norms, and relations and their importance to health outcomes in project design, implementation, and evaluation)?			
IF NO, THEN EXCLUDE			

8. Has the intervention been evaluated? (Is the evaluation described in the article?)			
IF NO, THEN EXCLUDE			
**Titles and abstracts (more detailed exclusionary questions)**			
Repeat questions 1–7			
9. Is the intervention gender-transformative (ie, does it meet at least one of the following criteria: 1) fostering critical examination of inequalities and gender roles, norms, and dynamics; 2) recognising and strengthening positive norms that support equality and an enabling environment; and 3) promoting the relative position of women, girls, and marginalised groups, and 4) transforming the underlying social structures, policies and broadly held social norms that perpetuate gender inequalities)?			
IF NO, THEN EXCLUDE			
10. Are the evaluation methods clearly identified?			
IF NO, THEN EXCLUDE			
11. Is the study quantitative or mixed-methods?			
IF QUANTITATIVE OR MIXED METHODS, PROCEED. IF QUALITATIVE ONLY, GO TO NUMBER 15			
12. Does the study use one of the following evaluation methodologies and/or statistical methods?<br>● Randomised controlled trial (RCT), including cluster RCTs<br>● Quasi-experiment (control and experimental group with no randomisation)<br>● Difference-in-difference (DD)<br>● Instrumental variables<br>● Matching<br>● Propensity score matching (PSM)<br>● Other matching			
IF NO, THEN EXCLUDE (OR GO TO NUMBER 15, IF MIXED METHODS)			
13. Does the study have a sample size of at least 50 people per treatment group for RCTs and at least 100 per treatment/control group for quasi-experimental methods at baseline?			
IF NO, THEN EXCLUDE (OR GO TO NUMBER 15, IF MIXED METHODS)			
14. Is the participant retention rate more than 60%?			
IF NO, THEN EXCLUDE (OR GO TO NUMBER 15, IF MIXED METHODS)			
15. Does the study include more than 30 individual in-depth interviews and/or at least 6 focus group discussions?			
IF NO, THEN EXCLUDE			
16. Are the sources appropriate for answering the described research questions/objectives?			
IF NO, THEN EXCLUDE			
17. Did at least two coders analyse the data?			
IF NO, THEN EXCLUDE			

Appendix:

Search terms

We searched electronic search engines including **EBSCO, ProQuest, Web of Science,** and **Scopus**. Within EBSCO, we searched databases using Academic Search Complete, America: History & Life, Applied Science and Technology Full Text, ATLA Religion Database, Business Source Complete, CINAHL Plus, Communication Abstracts, EconLit with Full Text, Family and Society Studies Worldwide, Gender Studies Database, Global Health, GreenFILE, Index to Legal Periodicals & Books Full Text, LGBT Life with Full Text, MEDLINE, Military & Government Collection, PsycINFO, social Work Abstracts, and SocINDEX with Full Text. Within ProQuest, we searched the following databases: Ethnic NewsWatch, GenderWatch, Index Islamicus, Literature on Traumatic Stress, ProQuest Social Sciences Premium Collection, and Social Science Premium Collection. Within Web of Science we used the Core Collection: Science Citation Index Expanded, Social Sciences Citation Index, Arts & Humanities Citation Index, and Emerging Sources Citation Index. Scopus is its own database.

We used the following set of search terms adapted for the specific syntax for each search engine:

TITLE-ABS ("babies" OR "baby" OR "infant" OR "infants" OR neonat* OR newborn* OR "nursery school*" OR "pre-school*" OR preschool* OR "pre school*" OR toddler* OR "underfive*" OR "under five" OR "under fives" OR kindergarten* OR "primary grade*" OR "first grade*" OR "second grade*" OR "third grade*" OR "fourth grade*" OR "fifth grade*" OR child* OR juvenile* OR "minor" OR "minors" OR youngster* OR underage* OR pediatric* OR "youth" OR "youths" OR "school age" OR "school-age" OR "school aged" OR "school-aged" OR "school based" OR "school-based" OR "grade school*" OR "elementary school*" OR "grammar school*" OR "elementary education" OR "middle school*" OR "puberty" OR "pre-adolescent*" OR preadolescent* OR "pre-teen" OR preteen* OR "pre-teens")) OR (AB,TI("pre-teenager*" OR "tween" OR "tweens" OR "sixth grade*" OR "seventh grade*" OR "eighth grade*" OR "high school*" OR "secondary school*" OR "secondary education" OR adolescen* OR "teen" OR "teens" OR teenager* OR "ninth grade*" OR "tenth grade*" OR "eleventh grade*" OR "twelfth grade*" OR "college student*" OR "university student*" OR "college age" OR "college-age" OR "college aged" OR "college-aged" OR "postsecondary" OR "post-secondary" OR "post secondary" OR "higher education" OR "emerging adult*" OR "early adulthood" OR "young adult*" OR "young person*" OR "young people" OR "young woman" OR "young women" OR "young man" OR "young men" OR "boy" OR "boys" OR "girl" OR "girls") AND TITLE-ABS (intervention* OR initiative* OR "project" OR "projects" OR "pilot" OR "program" OR "programme" OR "programs" OR "programmes" OR "programming" OR "trial" OR "trials" OR "curriculum" OR "curricula" OR experiment* OR "training" OR "workshop*" OR "inservice*" OR "in-service" OR "seminar" OR "seminars" OR "tutorial*" OR "campaign" OR "campaigns" OR simulat* OR "educational presentation*" OR "instructional presentation*" OR "educational session*" OR "instructional session*" OR treatment* OR "therapy" OR "therapies" OR "therapeutic*" OR rehab* OR psychotherap* OR "group work" OR "groupwork*" OR counselling OR counseling OR "self-help" OR "self help" OR "support group*") AND TITLE-ABS (effective* OR outcome* OR assess* OR evaluat* OR implement* OR impact* OR improve* OR benefit* OR beneficial* OR efficac* OR success* OR succeed* OR failed OR unsuccess* OR ineffective* OR qualitative* OR quantitative* OR compare* OR compari* OR random* OR "control group" OR "control groups" OR "systematic review*" OR "meta-analysis" OR "metaanalysis" OR "meta analysis" OR "metanalysis" OR "meta-analyses" OR "meta analyses" OR "metaanalyses" OR "metanalyses") AND TITLE-ABS ("gender* role*" OR "gender* identit*" OR "gender* attitud*" OR "gender* belief" OR "gender* norm*" OR "gender* stereotyp*" OR "gender* bias*" OR "gender* perception*" OR "gender* based" OR "gender-based" OR "gendered-based" OR "gender* behavio*" OR "gender* obstacle*" OR "gender* barrier*" OR "gender* relation*" OR "gender* dynamic*" OR "gender* inequalit*" OR "gender* inequit*" OR "gender* issue*" OR "gender* environment*" OR "gender* influen*" OR "gender* value*" OR "gender* imbalance*" OR "gender* disparit*" OR "gender* gap" OR "gender* gaps" OR "gender* unequit*" OR "gender* unequal*" OR "gender* unjust*" OR "gender* discriminat*" OR "gender* disadvantage*" OR "gender* exclusion*" OR "gender* injustice*" OR "gender* oppress*" OR "gender* prejudic*" OR "gender* empower*" OR "gender* disempower*")) OR TITLE-ABS ("gender*

disproportion*" OR "gender* marginali*" OR "gender* unfair*" OR "gender* challeng*" OR "gender constrain*" OR "gender* difficult*" OR "gender* hinder*" OR "gender* hindrance*" OR "gender* hurdle*" OR "gender* impedi*" OR "gender* impede*" OR "gender* limit*" OR "gender* obstruct*" OR "gender* opposition*" OR "gender* oppose*" OR "gender* opposing" OR "gender* problem*" OR "gender* roadblock*" OR "gender* road block*" OR "gender* stumbling block*" OR "gender* mores" OR "gender* integrat*" OR "gender* transform*" OR "gender* view*" OR "gender* perspective*" OR "gendered" OR "macho" OR machismo* OR marianismo* OR feminin* OR masculin* OR "sex role*")) OR (TITLE-ABS (gender* OR cisgender* OR "cis-gender*" OR transgender* OR "trans-gender*") AND (TITLE-ABS ("social environment*" OR "social influen*" OR "social value*" OR "socialization" OR "socialisation" OR "psychosexual development*" OR "stereotyp* behavio*" OR "social norm*" OR "social perception*" OR "social attitud*" OR "social belief*" OR "social bias*" OR "social behavio*" OR "social obstacle*" OR "social barrier*" OR "social inequalit*" OR "social* inequit*" OR "social* unequit*" OR "social* unequal*" OR "social* discriminat*" OR "social* disadvantage*" OR "social injustice*" OR "social* oppress*" OR "social* prejudi*" OR "social* empower*" OR "social* disempower*" OR "social* marginali*" OR "social* unfair*" OR "social* unjust*" OR "social* challeng*" OR "social* constrain*" OR "social* difficult*" OR "social* hinder*" OR "social hindrance*" OR "social hurdle*" OR "social* impedi*" OR "social* impede*" OR "social* limit*" OR "social* obstruct*" OR "social opposition*" OR "socially oppose*" OR "social road block*" OR "social roadblock*" OR "social stumbling block*" OR "social mores" OR "social view*") OR TITLE-ABS ("cultural norm*" OR "cultural value*" OR "cultural* bias*" OR "cultural perception" OR "cultural influen*" OR "cultural environment*" OR "cultural attitud*" OR "cultural belief*" OR "cultural behavio*" OR "cultural obstacle*" OR "cultural barrier*" OR "cultural inequalit*" OR "cultural* inequit*" OR "cultural* unequit*" OR "cultural* unequal*" OR "cultural* discriminat*" OR "cultural* disadvantage*" OR "cultural injustice*" OR "cultural* unjust*" OR "cultural* oppress*" OR "cultural* prejudi*" OR "cultural* empower*" OR "cultural* disempower*" OR "cultural* marginali*" OR "cultural* unfair*" OR "cultural* perspective*" OR "cultural view*" OR "cultural* challeng*" OR "cultural* constrain*" OR "cultural* hinder*" OR "cultural hindrance*" OR "cultural hurdle*" OR "cultural* impedi*" OR "cultural* impede*" OR "cultural* limit*" OR "cultural* obstruct*" OR "cultural opposition*" OR "cultural* oppose*" OR "cultural road block*" OR "cultural roadblock*" OR "cultural stumbling block*" OR "cultural mores") OR TITLE-ABS ("power imbalance*" OR "power differ*" OR "power relation*" OR "power dynamic*" OR "power attitud*" OR "power belief*" OR "power stereotyp*" OR "power bias*" OR "power perception*" OR "power inequalit*" OR "power inequit*" OR "power disparit*" OR "power unequit*" OR "power unequal*" OR "power discriminat*" OR "power disadvantage*" OR "power injustice*" OR "power oppress*" OR "power prejudi*" OR "power marginali*" OR "power unfair*" OR "power unjust*" OR "power view*" OR "power perspective*" OR "power constrain*" OR "power hinder*" OR "power hindrance*" OR "power hurdle*" OR "power impedi*" OR "power impede*" OR "power limit*" OR "power obstruct*" OR "power opposition*" OR "power oppose*")) AND TITLE-ABS (intervention* OR initiative* OR "project" OR "projects" OR "pilot" OR "program" OR "programme" OR "programs" OR "programmes" OR "programming" OR "trial" OR "trials" OR "curriculum" OR "curricula" OR experiment* OR "training" OR "workshop*" OR "inservice*" OR "in-service" OR "seminar" OR "seminars" OR "tutorial*" OR "campaign" OR "campaigns" OR simulat* OR "educational presentation*" OR "instructional presentation*" OR "educational session*" OR "instructional session*" OR treatment* OR "therapy" OR "therapies" OR "therapeutic*" OR rehab* OR psychotherap* OR "group work" OR "groupwork*" OR counselling OR counseling OR "self-help" OR "self help" OR "support group*") AND TITLE-ABS (effective* OR outcome* OR assess* OR evaluat* OR implement* OR impact* OR improve* OR benefit* OR beneficial* OR efficac* OR success* OR succeed* OR failed OR unsuccess* OR ineffective* OR qualitative* OR quantitative* OR compare* OR compari* OR random* OR "control group" OR "control groups" OR "systematic review*" OR "meta-analysis" OR "metaanalysis" OR "meta analysis" OR "metanalysis" OR "meta-analyses" OR "meta analyses" OR "metaanalyses" OR "metanalyses"))) AND (random* OR "rct*" OR "equivalence trial*" OR "quasi-experimental*" OR "quasiexperimental*" OR "quasi experimental*" OR "control group*" OR "controlled group*" OR "experimental group*" OR "experimental groups" OR "controlled trial" OR "controlled trials" OR "difference-in-difference*" OR "difference in difference*" OR "DID" OR "DD" OR "instrumental variable*" OR "IV" OR "matched-pair analysis" OR "matched-pair analyses" OR "matched pair analysis" OR "matched pair analyses" OR "paired comparison" OR "paired comparisons" OR "matching study" OR "matching studies" OR "propensity score" OR "propensity scores" OR "propensity scoring" OR "psm")).

Appendix 4:

Comprehensive search of the grey literature for gender-transformative programmes targeting 0–24 year olds

Our search for and inclusion of the grey literature began with an initial broad search of the internet using various combinations of the search terms used for the peer-reviewed literature. It became more intentional when we decided to identify key organisations and search their websites for documented evaluations of their programmes. This process would not only require an additional layer of communication with the identified organisations but also require the team to develop a structured plan for outreach and decision making.

To structure our search, we worked with the North Carolina-based consulting firm Iris Group, building on a task it had completed with the Bill and Melinda Gates Foundation (BMGF) to support gender capacity building within the organisation. For this task, Iris had collaborated with BMGF to develop a list of organisations doing gender-based work. This list included an initial group of 33 organisations, which were divided into five sectors: Development Banks, Multi-laterals, Bi-laterals, iNGOs, and Foundations. For each organisation, the Iris Group team examined organisation websites, strategy and policy documents, and tools and training materials. The team used this content to populate a grid briefly detailing each institution's geographic and sectoral focus, gender equality criteria, how and how long the criteria had been used, evaluations of the criteria, guidelines for their use, and an analysis of the criteria's relevance for BMGF. Based on these characteristics, the BMGF gender team selected 17 organisations for an in-depth examination of gender equality criteria.

Using this list of 17 organisations, we took the following steps:

1. Reviewed the grey literature that had already been collected and identified the organisations that were represented.
2. Compared the list of organisations represented in our initial search with the BMGF list of 17 organisations to see where there were gaps.
3. Reviewed the websites of the 'missing' organisations and downloaded relevant evaluations.

With regard to the funding organisations, the challenge was that many do not have robust sections on their websites for evaluation reports, nor do they systematically provide contact information for their grantees. After completing the three steps above, we shifted to sifting through the remainder of the organisations that were missing in a two-stage process (1: Google Scholar search; 2: website search).

Appendix: Coding for data extraction

COMPONENT	QUESTIONS	CODES
**A. IDENTIFYING CHARACTER-ISTICS**	A1. Author	
	A2. Date published	
	A3. Title of article	
	A4. Name of implementing organisation	
	A5. Name of programme, if applicable	
	A6. Funding source (list all that are mentioned for programme and/or evaluation)	
	A7. Was this programme assessed in multiple studies? If yes, does this evaluation tell us something new? If no, then exclude but write	If yes, provide reference for primary evaluation article
**B. PROGRAMME POPULATION**	B1. Age, in years, of participants/target population (Choose all that apply)	• 0–4<br>• 5–9<br>• 10–14<br>• 15–19<br>• 20–24<br>• 25–49<br>• 50+<br>• Other (Write in)
	B2. Biological sex of participants/target population (Choose all that apply)	• Girls<br>• Women (18+ years old)<br>• Boys<br>• Men (18+ years old)<br>• Girls and boys together<br>• Women and men together<br>• Girls and boys separately<br>• Women and men separately<br>• Everyone together<br>• Not clear<br>• Other/does not apply (Describe)
	B3. Marginalised/specific groups (Choose all that apply)	• Affected by conflict<br>• Black/African American<br>• Community leaders<br>• Disability<br>• Domestic workers<br>• Ethnic/religious minority<br>• Family members/in-laws<br>• Indigenous<br>• Latinx<br>• Lesbian, gay, bisexual, transgender, queer or questioning, and intersex (LGBTQI)<br>• Marginalised caste<br>• Married girls<br>• Married men<br>• Newly wedded couples<br>• Orphans<br>• Out-of-school girls<br>• Out-of-school boys<br>• Parents<br>• Post-secondary school<br>• Recent migrants<br>• Religious leaders<br>• Sex workers<br>• Teachers<br>• Unmarried girls/women<br>• Very poor<br>• None specified<br>• Other (Write in)

	B4. Are the program's participants/*target population* the same as the *beneficiary population?*<br><br>**Example:** A school-based programme that trains teachers in gender norms to improve students' secondary school attrition rates. The *target population* would be the teachers, and the *beneficiary population* would be the students.	• Yes, they are the same (Skip to section 'D')<br>• No, they are different (Continue)
	B5. Age, in years, of beneficiary population (Choose all that apply)	• 0–4<br>• 5–9<br>• 10–14<br>• 15–19<br>• 20–24<br>• 25–49<br>• 50+<br>• Other (Write in)
	B6. Biological sex of beneficiary population (Choose all that apply)	• Girls<br>• Women (18+ years old)<br>• Boys<br>• Men (18+ years old)<br>• Girls and boys<br>• Women and men<br>• All<br>• Not clear<br>• Other/does not apply (Describe)
**C. GEOGRAPHY & CONTEXT**	C1. Context	• Rural<br>• Urban<br>• Peri-urban<br>• Not specified
	C2. Country/ies	• Fill in blank
	C3. Region (Choose all that apply)	• East Asia and Pacific<br>• Europe and Central Asia<br>• Latin America and Caribbean<br>• Middle East and North Africa<br>• North America<br>• South Asia<br>• Sub-Saharan Africa<br>• Oceania
**D. PROGRAMME INFORMATION**	D1. Health/development sector (Choose all that apply)	• Family planning/reproductive health<br>• HIV<br>• Infectious disease<br>• MCH<br>• Mental health<br>• Multisectoral<br>• Non-communicable disease<br>• Nutrition<br>• Physical activity<br>• Substance abuse<br>• Unintentional injury<br>• Violence<br>• WASH<br>• Other (Describe)
	D2: Type of programme (Choose all that apply)	• Education/skills training<br>• Community events (eg, theatre, health fairs)<br>• Social marketing and info/awareness campaigns<br>• Institutional capacity building (eg, school, workplace)<br>• Other (Describe)

	D3. Programme activities (Choose all that apply)<br><br>Using a social determinants of health framework	**Related to economic stability**<br>• Financial literacy education<br>• Savings or loans<br>• Stipends or incentives<br>**Related to neighbourhood/physical environment**<br>• Safe space/Space to relax, socialise, and build social networks<br>• Sports<br>• Local health fairs<br>• Theatre/drama<br>• Prayer/religious meetings/messages<br>**Related to general education/training**<br>• Communication skills training<br>• Literacy training<br>• Language training<br>• Early childhood education<br>• Vocational training<br>• Higher education<br>• Health information education<br>• Education about rights/the law<br>• Gender norm education<br>**Related to community and social context**<br>• Social integration/life skills training<br>• Provision of support system<br>• Community engagement<br>• Political/civic engagement<br>**Health care system**<br>• Health coverage<br>• Provider availability/training<br>• Changing gender-related discriminatory attitudes and policies<br>• **Unclear**<br>• **None**
	D4. Programme methodology	• Dialogue/discussion sessions<br>• Role play<br>• Peer-to-peer<br>• Print materials<br>• Video/radio/TV<br>• Computer- or phone-based learning<br>• Unclear
	D5. Where programme takes place<br><br>Health care settings should not be included in this review unless they are in conjunction with another implementation strategy.	• Community<br>• Health care setting<br>• Prison system<br>• Religious setting<br>• School-based (eg, as part of school curriculum)<br>• School extracurricular (eg, after school)<br>• Workplace<br>• Combination
	D6. Who was involved in developing/implementing the programme	• NGO<br>• Government<br>• Religious organisation<br>• Private sector<br>• Membership organisation/association<br>• University/research institution<br>• UN agency<br>• Unclear
	D7. Overall length of programme (period over which programme was implemented)	• Less than one week<br>• 1 week – <3 months<br>• 3 – <6 months<br>• 6 – <12 months<br>• 12 – <24 months<br>• Over 24 months<br>• Unspecified/unclear

	D8. Number of contact encounters	• 1 time<br>• 2–4 times<br>• 5–10 times<br>• 11–20 times<br>• 21+ times<br>• Unspecified/unclear
	D9. Frequency of contact encounters	• More than once a week<br>• Once a week<br>• Less than once a week but more than once a month<br>• Once a month<br>• Irregularly or less frequently<br>• Unspecified/unclear
	D10. Scale of programme (number of participants)	• Fewer than 200 participants<br>• 200 – <1 000<br>• 1 000 – <10 000<br>• 10 000 – <50 000<br>• More than 50 000<br>• Unclear
	D11. Do programme participants pay to participate?	• Yes<br>• No<br>• Unclear
	D12. Do programme participants receive payment/incentives to participate?	• Yes<br>• No<br>• Unclear
**E. OUTCOMES**	E1: Specific health outcome(s) measured:<br><br>Note whether there was a positive change, negative change, or no change, and whether it was statistically significant, non-significant, or N/A. If an outcome is not listed here, please add it to the database.	**Knowledge/attitudes**<br>• Birth knowledge and preparedness<br>• Body satisfaction<br>• Family planning methods and use<br>• Sexual and reproductive health (SRH)<br>• STI prevention and/or service availability<br>• Stigma reduction<br>**Behaviours**<br>• Abortion/unsafe abortion<br>• Aggression<br>• Birth preparedness<br>• Body comparison<br>• Breastfeeding practices<br>• Contraceptive use/continuation<br>o Condom use<br>o Long acting reversible contraceptive use<br>o Modern contraceptive use<br>o Male sterilisation<br>o Female sterilisation<br>• Diarrhoea management<br>• Dietary restraint<br>• Exercise<br>• Gender-based violence<br>• Interpersonal communication about:<br>o HIV<br>o STIs (not HIV)<br>o Family planning<br>• Nutritious eating<br>• Risky sexual behaviour (number of partners/unprotected sex)<br>• Sanitation and hygiene practices<br>• Sexual behaviour<br>• Smoking cessation<br>• Substance abuse<br>**Access to/use of services**<br>• ARV treatment<br>• Contraceptive services<br>• HIV testing/screening<br>• Mammograms

		• Pap smears<br>• Prenatal/antenatal care visits<br>• Postpartum care visits<br>• STI screening<br>• Treatment for STIs<br>• Use of a skilled birth attendant<br>• Vaccinations<br>**Other health outcomes**<br>• Anorexia<br>• Anxiety<br>• Bulimia<br>• Child morbidity (age >2 years)<br>• Depression<br>• DSM conduct disorder<br>• DSM ADHD<br>• Gun safety<br>• HIV status<br>• Infant mortality<br>• Infant morbidity<br>• Low birth weight<br>• Maternal mortality<br>• Maternal morbidity<br>• Menstrual hygiene<br>• Pregnancy (incidence)<br>• Preterm birth<br>• PTSD<br>• Safety<br>• Social problems<br>• Suicidality<br>• Unwanted/unintended pregnancy
	E2: Gender-related outcome(s) measured<br><br>Note whether there was a positive change, negative change, or no change, and whether it was statistically significant, non-significant, or N/A. If an outcome is not listed here, please add it to the database.	**Social and psychological empowerment**<br>• Ability to negotiate desires<br>• Changes in self-confidence<br>• Changes in aspirations<br>• Changes in ability to take action/self-efficacy<br>• Civic/political engagement<br>• Experience of leadership/leadership skills<br>• Family relationships<br>• Partner communication<br>• Social relationships<br>**Changes in knowledge related to:**<br>• Available services<br>• Finance<br>• Law<br>• Rights<br>**Changes in attitudes related to:**<br>• Gender norms (ie, what is expected of girls versus boys)<br>• Mobility/freedom of movement<br>• Child marriage/age at marriage<br>• Violence<br>• FGM/C<br>• Division of domestic care/work<br>**Changes in practices**<br>• Age at marriage or rate of child marriage<br>• FGM/C<br>• Experience of physical or sexual violence<br>• Experience in mobility<br>• Domestic divisions of labour<br>• Increased male involvement in health-related behaviours<br>**Changes in economic wellbeing**<br>• Ability to save/access to savings<br>• Access to credit<br>• Banking services<br>• Control over assets

		• Income generation<br>• Self-employment<br>• Financial literacy<br>• Ownership of land<br>**Change in access to/use of services**<br>• Health services<br>• Education services<br>• Educational achievement<br>**Changes in social structures, laws, policies, or systems**
	E3. Significant changes in health and gender outcomes<br><br>**Note:** These changes are by default understood to be good (whether they increase or decrease). If the changes were detrimental, but still significant, then please describe that in the info.	• Significant change in health-related outcome, significant change in gender-related outcomes<br>• Sig change in health, non-sig chance in gender<br>• Non-sig change in health, sig change in gender<br>• Non-sig change in health, non-sig change in gender
	E4. Evidence of spillover/contamination<br><br>**Remember:** *Spillover* is when information from programme 'spills over' from experimental group to control group, and *contamination* is when the groups are influenced by outside programmes. Both lead to biased results.	• Yes, evidence of spillover effects<br>• No evidence of spillover effects<br>• Spillover not discussed<br>• Yes, evidence of contamination<br>• No evidence of contamination<br>• Contamination not discussed
	E5. Evidence about cost-effectiveness	• Evidence on cost-effectiveness provided<br>• Not discussed/provided
	E6. Evidence on long-term effects	• Evidence on long-term effects provided<br>• Not discussed/provided
**F. EVALUATION INFO AND METHODS**	F1. Design type	• Quantitative only (Proceed to next question)<br>• Mixed methods (Proceed and answer questions that are applicable)
	F2. Quantitative design	• Randomised controlled trial (RCT), including cluster RCTs<br>• Quasi-experiment (pre-test, post-test with controls)<br>• Other (Describe, fill in blank)<br>○ Pre-test, post-test no control<br>○ Post-test only with control groups
	F3. Quantitative analytical techniques	• Difference-in-difference (DD)<br>• Instrumental variables<br>• Matching<br>• Propensity score matching (PSM)<br>• Synthetic controls<br>• Other (Describe, fill in blank)
	F4. Qualitative methods<br><br>Applicable only for mixed methods with qualitative component	• In-depth interviews<br>• Focus groups<br>• Participatory research<br>• Photovoice<br>• Semi-structured interviews<br>• Key informant interviews
	F5. Sample selection for qualitative designs<br><br>Applicable only for mixed methods with qualitative component	• Convenience sample<br>• Snowball sampling<br>• Theoretical and/or intentional sampling<br>• Unclear<br>• Other (Describe)
	F6. Who conducted the evaluation?	• Internal team (employees of programme)<br>• External evaluators (not involved in programme implementation)<br>• Mixed internal and external team<br>• Unclear

	F7. Length of evaluation (period of programme time considered by evaluation)	• 0 – <3 months<br>• 3 – <6 months<br>• 6 – <12 months<br>• 1 – <3 years<br>• 3 – <5 years<br>• Over 5 years
	F8. When evaluation was conducted	• While programme was being implemented<br>• 0–6 months after programme/participation<br>• 7–12 months after programme/participation<br>• 1–2 years after programme/participation<br>• 3–4 years after programme/participation<br>• 5+ years after programme/participation<br>• Not specified
	F9. Retention rate	• >80%<br>• 60–80%<br>• <60%<br>• Not clear
	F10. Sample size (ie, size of the experimental group)	• 50–100<br>• 101–300<br>• 301–1 000<br>• >1 000
	F11. Overall reliability of evaluation	• High<br>• Medium<br>• Low

*The development of these codes draws on the previous experience of, and coding techniques for, creating synthesis reports at the Overseas Development Institute (ODI).

Appendix:
Characteristics of High-Quality Gender-Transformative Programmatic Interventions

Using the 'Levy-Green Tool' below, programmes were further categorised as high-quality if they demonstrated the following evidence of leading to large-scale norm change and sustained improvements in health:

(1) Multiplicity: Affected outcomes beyond the specific health area of focus.

(2) Sustainability: Demonstrated measured change at the individual, community, and/or institutional level at least one year after programme completion, holding promise for lasting improvements in health and gender equality.

(3) Spreadability: Addressed discriminatory gender-related attitudes and behaviours that harm health by either directly or indirectly spreading the change/outcome to individuals outside the programme.

(4) Scalability: Had been, or was poised to be, expanded or replicated to cover a larger geographic region or population.

**Levy-Greene Tool: Identifying Evidence of Impact**			
**Multiplicity**	**Sustainability**	**Spreadability**	**Scalability**
Affects outcomes beyond the specific health outcome of focus	*Change is sustained in exposed individuals and/or community/institution*	*Change/Outcome is passed to individuals outside the interventions either directly or indirectly*	*Expanding or replicating interventions with the aim of covering a larger geographic region and/or population*
● Behaviors and outcomes in multiple health and development areas are measured and improved	● Individuals sustain measured behavior/outcome at least one year after end of intervention<br>● Measured change in structural social determinants	● Measures existence/extent of social networking and spread<br>● Measures intergenerational / intrafamilial effects<br>● Multilevel intervention	● Has already been brought to scale<br>● Is being piloted in an institution within which there is a plan for scaleup<br>● Implementers make a strong case for ability to be brought to scale

Appendix:

Data extraction for gender-transformative programmes included in review

Tables are sorted by area of health:

1. Family planning and sexual & reproductive health
2. HIV
3. Maternal & child health
4. Violence
5. Multiple areas of health

Articles are sorted by programme name; those that were characterized as high quality are highlighted.

Table 1: **Family planning and sexual & reproductive health**

Programme	Programme Description	Illustrative Evidence - Health	Illustrative Evidence - Gender
**African Youth Alliance**<br><br>Karim et al.<br>2009<br><br>*Uganda*	*Goal:* to improve sexual and reproductive health (SRH) and reduce STI transmission<br><br>*Participants:* girls and boys aged 10–24, teachers, healthcare providers, social workers, parents, religious leaders, the media, politicians, and policy makers<br><br>*Programme Components:*<br>• Policy and advocacy coordination<br>• Best-practices coordination and dissemination<br>• Institutional capacity building<br>• Youth-friendly SRH services<br>• Behaviour-change communication	*Statistically Significant Effects:*<br>• Condom and contraceptive use for both girls and boys<br>• Fewer sexual partners, increased condom and contraceptive use among girls<br>• Initial sexual experience after age 16 among boys	*Statistically Significant Effects:*<br>• Decreased engagement in risky sex
**Berhane Hewan**<br><br>Erulkar & Muthengi<br>2009<br><br>*Ethiopia*	*Goal:* to decrease child marriage and provide support for already married girls through building their social, health, and economic assets<br><br>*Participants:* married and unmarried girls aged 10–19<br><br>*Programme Components:*<br>• Support for girls to stay in school or non-formal and livelihood training for out-of-school girls<br>• Formation of girls' groups with female mentors<br>• Community conversations to discuss key issues re: child marriage and encourage collective problem solving	*Statistically Significant Effects:*<br>• Awareness of contraceptives (pill, injectable contraceptives, and condoms)<br>• HIV/AIDS knowledge<br>• Communication with close friends about HIV/AIDS, STIs, family planning methods, condoms, violence in community and problems in their marriage if married<br>• Use of contraceptives	*Statistically Significant Effects:*<br>• Decreased likelihood to have ever been married<br>• Increased likelihood to be currently in school, to read easily, and to not be married<br>• Decreased likelihood to discuss with their friends what type of spouse they desired
**CHOICES**<br><br>Institute for Reproductive Health, Georgetown University<br>2013<br><br>*Nepal*<br>*(Siraha District)*	*Goal:* to empower girls and boys to challenge and change inequitable gender norms and corresponding behaviours in their communities that affect educational attainment, livelihoods, and health through dialogue<br><br>*Participants:* girls and boys aged 10–14 who are currently enrolled in Save the Children's child clubs<br><br>*Programme Components:*<br>• Eight 2-hour discussion groups that covered gender equity, interpersonal communication, and risks for girls and women (e.g. violence and child marriage)	*Qualitative Findings:*<br>• Boys reported speaking with friends and neighbours about girls' education and delaying age at marriage<br>• Decreased teasing among youth	*Statistically Significant Effects:*<br>• Fewer youth felt it was acceptable for a man to beat his wife over a disagreement<br>• Increased number of youth felt that girls should have the same chances as boys to go to school or work outside the home<br>• Increased gender-equitable attitudes (eg, division of household labour, gender roles, power)<br><br>*Qualitative Findings:*<br>• Girls reporting their brothers helping with household chores<br>• Decreased ridicule of boys and men who help with traditionally female tasks<br>• Increased respect among female and male siblings<br>• Parents expressed more equitable aspirations for daughters and sons

Programme	Programme Description	Illustrative Evidence - Health	Illustrative Evidence - Gender
**Development Initiative Supporting Healthy Adolescents (DISHA)**<br><br>Kanesathasan et al. 2008<br><br>*India (Bihar and Jharkhand)*	*Goal:* to improve SRH outcomes for girls and boys by addressing social and economic influences<br><br>*Participants:* girls and boys aged 10–24, parents, SRH service providers, NGOs, general community members<br><br>*Programme Components:*<br>• Youth groups and resource centres that provide health information on SRH topics (eg, adolescence, gender, sexuality, fertility awareness, contraception, HIV/AIDS, safe motherhood)<br>• A trained cadre of peer educators<br>• Livelihoods training for youth<br>• Community awareness media campaign<br>• Youth contraceptive depots and youth-friendly training for providers	*Statistically Significant Effects:*<br>• Increased knowledge of how to access the pill among married girls<br>• Increased knowledge of condoms among girls and boys<br>• Decreased number of girls who disapproved of contraceptive use among married couples<br>• Increased number of girls and boys who believed that contraceptives should be available to young married couples	*Statistically Significant Effects:*<br>• Increased percentage of girls and boys who knew the legal age of marriage for girls<br>• Increased odds that unmarried girls and boys were able to talk with their elders about marriage timing<br>• Increased reported ability of girls to access SRH services unaccompanied<br><br>*Non-significant Effects:*<br>• Increased ability of married girls to speak with spouse about contraception
**Employment and Livelihood for Adolescents Centres**<br><br>Shahnaz & Karim 2008<br><br>*Bangladesh*	*Goal:* to create opportunities for girls to gain control over their lives and promote their own wellbeing through social and financial empowerment<br><br>*Participants:* girls aged 10–24<br><br>*Programme Components:*<br>• Microfinance with weekly meeting to pay loan and savings instalments<br>• Skill-based training on income-generating activities (IGA)<br>Weekly meetings where girls could gather to play games, exchange books, socialise, and have topic-based discussions (e.g. health, child marriage, dowry, girls' rights, responsibility to family and society)	*Non-significant Effects:*<br>• Knowledge of HIV/AIDS and STIs and routes of transmission	*Statistically Significant Effects:*<br>• Increased self-reports of reading non-textbook materials in the last month<br>• Higher mean score of writing<br>• Increased perceived mobility<br>• Receipt of income generation trainings<br>• Involvement in IGAs<br>• Higher average earnings in the last 6 months<br><br>*Non-significant Effects:*<br>• Financial literacy<br>• Awareness of legal age or marriage<br>• Awareness of consequences of child marriage
**Gender Matters (GEN.M)**<br><br>Smith et al. 2016<br><br>*United States (Texas)*	*Goal:* to reduce teenage pregnancy and related sexual risk behaviours by challenging gender roles and promoting healthy relationships<br><br>*Participants:* girls and boys aged 14–16<br><br>*Programme Components:*<br>• 5-day, 20-hour curriculum-based educational workshop covering gender, healthy relationships, decision making, and pregnancy prevention<br>• 4-month social media campaign that reinforces workshop messages<br>• Community film screening of participants' reflections on workshop topics	*Statistically Significant Effects:*<br>• Knowledge of where to obtain female birth control methods<br><br>*Non-significant Effects:*<br>• Youth attitudes towards pregnancy and contraception<br>• Youth intentions related to sex and contraceptive use<br>• Youth sexual activit	*Non-significant Effects:*<br>• Youth gender role attitudes<br>• Perceived refusal skills

Programme	Programme Description	Illustrative Evidence - Health	Illustrative Evidence - Gender
**Kishori Abhijan**<br><br>Amin et al.<br>2005<br><br>*Bangladesh*	*Goal:* to empower girls to have control over consequential decisions in their lives through the provision of life and livelihood skills<br><br>*Participants:* girls aged 13–22<br><br>*Programme Components:**<br>• Adolescent Organized Peer Network (APON)<br>• Provision of education on health, legal rights, early marriage, and dowry; group games; access to books<br>• Junior Village Organization (APON/JVO)<br>• APON programme plus microcredit for qualified participants<br>• CMES<br>• Microcredit combined with education and awareness<br><br>**Each programme was implemented separately in 3 different villages*	*Statistically Significant Effects:*<br>• Awareness of STIs	*Statistically Significant Effects:*<br>• Likelihood of staying in school<br>• Likelihood of working for pay<br>• Changes in attitudes towards gender norms<br>• Changes in attitudes towards violence<br>• Changes in attitudes towards division of domestic care
**Sexuality Education Initiative (SEI)**<br><br>Constantine et al.<br>2015<br><br>*United States (California)*	*Goal:* to improve the SRH of high school students through comprehensive education and service provision<br><br>*Participants:* high school students, parents<br><br>*Programme Components:*<br>• 12-session classroom curriculum for 9th-grade students addressing gender roles, power dynamics in relationships & media messages, sexual rights, sexual & reproductive anatomy, pregnancy, STIs/HIV, and contraception<br>• After-school peer advocate programme training students to plan SRH awareness events and provide information on SRH services available at school<br>• Provision of sexual health services on school grounds<br>• Educational workshops and materials for parents	*Statistically Significant Effects:*<br>• Increased knowledge about sexual health and sexual health services<br><br>*Non-significant Effects:*<br>• Increased communication with sexual partners<br>• Increased intentions to use condoms	*Statistically Significant Effects:*<br>• More positive attitudes about sexual relationship rights<br>• Greater self-efficacy to manage risky situations
**SUUBI Research Program**<br><br>Ssewamala et al.<br>2010<br><br>*Uganda (Rakai District)*	*Goal:* to improve the health and life outcomes of AIDS-orphaned adolescents through microfinance<br><br>*Participants:* primary school-aged students who have lost at least 1 parent to AIDS<br><br>*Programme Components:*<br>• Twelve 1–2 hour economic educational workshops over 10 months covering asset building, saving, and small business development<br>• Monthly mentorship programme with peer mentors about planning for the future<br>• A 2:1 matched child savings account to pay for post-primary education, into which anyone could pay (eg, family, employers) but was held in child's name<br>• **Both intervention and control groups received the usual care for AIDS-orphaned children: counselling and provision of education-related supplies (eg, textbooks)*	*Statistically Significant Effects:*<br>• Youth reported lower acceptance of sexual risk-taking behaviours<br>• Positive change in boys' attitudes toward risk-taking behaviours<br><br>*Non-significant Effects:*<br>• Girls' attitudes toward risk-taking behaviours	*Non-significant Effects:*<br>• Girls' and boys' saving rates<br>• Average monthly net deposits

Programme	Programme Description	Illustrative Evidence - Health	Illustrative Evidence - Gender
**Tap and Reposition Youth (TRY)**<br><br>Erulkar & Chong 2005<br><br>*Kenya (Nairobi)*	*Goal:* to reduce girls' vulnerability to negative social and reproductive health outcomes through improving livelihood options<br><br>*Participants:* out-of-school girls aged 16–22 residing in low-income and slum areas<br><br>*Programme Components:*<br>• Microfinance groups that register as self-help groups and open a savings account in group's name<br>• Minimum required savings of 50 KSH per week per member<br>• 10,000 KSH loans distributed to members on a rolling basis, determined by groups votes on the strength of their business plans<br>• 6-day training on business management and reproductive health<br>• Weekly meetings for business advice and serve as a source of social support<br>• Mentorship programme with adults from various professions; activities depend on groups' expressed needs	*Statistically Significant Effects:*<br>• Knowledge that a healthy person can have HIV<br>• Ability to refuse sex<br>• Ability to insist on condom use during sex<br>• Ability to take part in decision to use condoms<br>• Knowledge that pills do not cause infertility<br>• Knowledge that there is no cure for HIV	*Statistically Significant Effects:*<br>• Number of women with household assets >7<br>• Number of women with savings<br>• Number of women who kept their savings in the bank<br>• Higher mean weekly earning<br>• Likelihood to disagree that women should not be able to refuse her husband sex<br>• Likelihood to disagree that it is necessary to have a husband to be happy
**Vietnamese Focus on Kids & Exploring the World of Adolescents**<br><br>Pham et al. 2012<br><br>*Vietnam (Ha Noi & Khanh Hoa Province)*	*Goal:* to improve SRH through education and youth-friendly services<br><br>*Participants:* unmarried girls and boys aged 15–20, parents, healthcare providers<br><br>*Programme Components:**<br>• Vietnamese Focus on Kids (VFOK)<br>• 10 weekly 2-hour sessions covering a variety of topics including puberty, HIV/AIDS, STIs, pregnancy, contraception, communication, condom use, and decision-making skills for girls and boys<br>• Exploring the World of Adolescents (EWA)<br>• 2 distinct curricula with 10 weekly 2-hour sessions, tailored to gender-specific contexts and needs, covering similar topics as VFOK, gender norms, and social constructs, that are and focus on gender norms and social constructs<br>• Exploring the World of Adolescents Plus (EWA+)<br>• 10 weekly 2-hour sessions for girls and boys<br>• Six 2-hour sessions for parents focusing on parent-child communication<br>• Two-day workshop for healthcare providers covering medical knowledge of SRH issues, adolescent development, and skills for communicating with adolescents<br><br>**Each programme was implemented separately in 3 different communities*	*Statistically Significant Effects:*<br>• Knowledge of pregnancy and contraception (EWA, EWA+)<br>• Knowledge of HIV/AIDs and STIs (EWA, EWA+, VFOK)<br><br>*Non-significant Effects:*<br>• Perceived severity of pregnancy<br>• Perceived severity of HIV/AIDS<br>• Perceived vulnerability to sex and HIV/AIDS<br>• Response efficacy<br>• Response cost	*Statistically Significant Effects:*<br>• Self-efficacy for condom use<br>• Self-efficacy for abstinence

Programme	Programme Description	Illustrative Evidence - Health	Illustrative Evidence - Gender
**Intervention Research**<br><br>Sebastian et al. 2004<br><br>*India (Allahabad)*	*Goal:* to increase girls' agency and decision-making power through life and livelihood skills<br><br>*Participants:* girls aged 14–19<br><br>*Programme Components:*<br>• 7–10 weekly SRH education group lessons*<br>• 19 vocational training courses (ranging in length from 1–2 weeks to several months) offered over the following 10 months<br>• Assistance opening saving accounts<br><br>**Received by both intervention and control groups*	*Statistically Significant Effects:*<br>• Improved reproductive knowledge	*Statistically Significant Effects:*<br>• Increased social skills index scores<br>• Increased self-reported hours of leisure time<br>• Greater number of girls reporting group membership<br>• Increased knowledge of safe spaces<br><br>*Non-significant Effects:*<br>• Increased gender role and self-esteem index scores<br>• Freedom of mobility to visit relatives without accompaniment<br>• Increased hours participating in the labour market

***Table 2:* HIV**

Programme	Programme Description	Illustrative Evidence - Health	Illustrative Evidence - Gender
**Biruh Tesfa**<br><br>Erulkar et al. 2013<br><br>*Ethiopia*	*Goal:* to reduce HIV infection among girls through building social support and prevention-related skills<br><br>*Participants:* very poor out-of-school girls aged 10–19<br><br>*Programme Components:*<br>• Formation of girls' groups by trained female adult mentors<br>• 30-hour educational curriculum, covering topics such as self-esteem, communication, gender and power dynamics, rape and coercion, menstruation, reproductive anatomy, STIs, HIV/AIDS, voluntary counselling and testing (VCT), ART, financial literacy, and basic literacy, delivered at group meetings	*Statistically Significant Effects:*<br>• Knowledge that there was no cure for AIDS<br>• Knowledge that a woman can give HIV to her unborn baby<br>• Knowledge of where to get VCT<br>• Desire to have VCT	*Statistically Significant Effects:*<br>• Likelihood of knowing a person from whom she can borrow money if needed urgently<br>• Likelihood to have at least one social safety net
**Empowerment & Livelihood for Adolescents (ELA)**<br><br>Bandiera et al. 2012<br><br>*Uganda*	*Goal:* to prevent HIV infection among girls through reducing sexual risk behaviours and economic vulnerability<br><br>*Participants:* girls enrolled in school full-time<br><br>*Programme Components:*<br>• Creation of Adolescent Development Clubs that are open 5 afternoons a week and provide life skills and vocational training programmes led by female mentors<br>• Life skills training sessions cover SRH, menstruation, pregnancy, STIs, HIV/AIDS, family planning, rape, management skills, conflict resolution, and leadership<br>• Vocational training, taught by entrepreneurs, includes courses on income-generating activities and emphasises establishing small-scale enterprises	*Statistically Significant Effects:*<br>• Knowledge of HIV<br>• Knowledge of pregnancy<br>• Knowledge of condom use<br>• Decreased likelihood of having sex unwillingly	*Statistically Significant Effects:*<br>• Greater Gender Empowerment Index scores<br>• Entrepreneurial ability<br>• Engagement in any income-generating activities<br>• Self-employment<br>• Satisfaction with income
**Primary School Action for Better Health (PSABH)**<br><br>Maticka-Tyndale et al. 2007<br><br>*Kenya (Nyanza Province)*	*Goal:* to reduce risk of HIV infection through delaying sexual debut, decreasing sexual activity, and increasing condom use<br><br>*Participants:* upper primary school students aged 11–16<br><br>*Programme Components:*<br>• Comprehensive in-service training on sexuality and HIV prevention programme for primary school teachers and student peer supporters<br>• Teachers train colleagues on integrating HIV education into all subjects<br>• Integration of sexuality and HIV education curriculum into existing school curriculum while using diverse co-curricular activities	*Statistically Significant Effects:*<br>• Students 3 times more likely to report 'high levels' of exposure to HIV and AIDS education<br>• Increased HIV and AIDS knowledge among boys who were virgins prior to programme implementation<br>• Increased condom use at last intercourse among boys<br><br>*Qualitative Findings:*<br>• Gains in knowledge across all groups<br>• Boys and girls with and without history of sexual intercourse responded differently for establishing alternative 'scripts'	*Statistically Significant Effects:*<br>• Increased self-efficacy among girls sexually active before programme implementation, as demonstrated by greater likelihood of reporting they:<br>• Could 'say "no" to sex'<br>• Could 'have a boyfriend and not play sex'

Programme	Programme Description	Illustrative Evidence - Health	Illustrative Evidence - Gender
**Sex & Risk**<br><br>Petersen et al.<br>2004<br><br>*South Africa (Durban)*	*Goal:* to reduce sexual risk behaviours and HIV risk through participatory education and awareness of social influences on health behaviours<br><br>*Participants:* 1st-year university students<br><br>*Programme Components:*<br>• Over 8 small group sessions, 3 content areas are covered:<br>• HIV/AIDS knowledge<br>• Critical consciousness (ie, self-reflection on gender norms and social pressures on sexual behaviours)<br>• Skill building (eg, condom use and negotiation)<br><br>**Control group received standard HIV/AIDS curriculum taught at the university*	*Statistically Significant Effects:*<br>• General HIV/AIDS knowledge<br>• Increased transmission knowledge<br><br>*Qualitative Findings:*<br>• Experience of 'AIDS information fatigue' and fear of becoming infected with HIV contributed to participants' lack of interest in the program	*Statistically Significant Effects:*<br>• Understanding of social influences among males<br>• Intention to resist negative influences among males<br><br>*Non-significant Effects:*<br>• Self-efficacy in condom use<br>• Assertive communication
**SHERO's Program**<br><br>Harper et al.<br>2009<br><br>*United States*	*Goal:* to prevent HIV infection through recognition and reduction of risk behaviours<br><br>*Participants:* Mexican-American girls aged 12–21<br><br>*Programme Components:*<br>• Nine 2-hour group sessions that cover a variety of topics through discussions, lectures, role plays and games:<br>• Risk reduction strategies (eg, condom negotiation)<br>• Gender-based inequities (eg, GBV, IPV, power dynamics)<br>• Social norms that impact SRH (eg, cultural pressures to be a mother, age-disparate sexual partners)<br><br>**Control group received a single 2-hour HIV/AIDS prevention program*	*Statistically Significant Effects:*<br>• More positive attitudes towards condoms<br>• Increased beliefs that peers support protective sexual behaviours<br>• Increased HIV/AIDS and STI prevention knowledge<br>• Increased reported condom use<br><br>*Non-significant Effects: at 2-Month Follow-Up*<br>• Less likely to report having vaginal sex<br>• More likely to carry condoms<br>• Intention to use condoms during the next 2 months	*Statistically Significant Effects:*<br>• Self-esteem<br>• Decrease in beliefs supporting violence against sexual partners<br>• Increased communication skills to discuss health with a sexual partner
**Somos Diferentes, Somos Iguales (SDSI)**<br><br>Solórzano et al.<br>2008<br><br>*Nicaragua*	*Goal:* to prevent HIV infection by addressing social and cultural barriers to prevention<br><br>*Participants:* girls and boys aged 10–25<br><br>*Programme Components:*<br>• National TV series ('Sexto Sentido') and a nightly youth talk call-in radio show that incorporate sexuality issues<br>• Development and distribution of support materials, resources, and campaign materials for use by local groups<br>• Media campaigns and workshops that focus on HIV prevention in the context of young people's sexuality, gender norms (machismo as a HIV risk factor), risk perception, decision making, and the power of speaking about taboo subjects<br>• Leadership training for youth leaders to strengthen analytical and leadership skills related to SRHR issues, including a focus on gender, stigma, discrimination, and GBV	*Statistically Significant Effects:*<br>• Increased knowledge and use of HIV- and IPV-related services<br>• Increased likelihood of consistent condom use with casual partners<br>• Increased interpersonal communication about domestic violence, HIV, homosexuality, condom use, and the rights of young people<br><br>*Non-significant Effects:*<br>• Condom use with steady partners	*Statistically Significant Effects:*<br>• Higher scores on gender-equitable attitudes associated with greater exposure (ie, watched at least 2 seasons) to SDSI<br>• Less stigmatising attitudes towards homosexuality an people living with HIV associated with greater exposure to SDSI<br>• Higher perceived self-efficacy to negotiate condom use<br>• Increased partner communication about HIV prevention

Programme	Programme Description	Illustrative Evidence - Health	Illustrative Evidence - Gender
	• Alliance building and coordination among young people and adults across local non-profits, health and social service providers, journalism and media outlets, and coalitions concerned with SRHR, human rights, women's issues, and violence		
**Intervention Research**<br><br>Visser<br>2007<br><br>*South Africa (Tshwane)*	*Goal:* to prevent HIV infection through reducing sexual risk behaviours<br><br>*Participants:* Secondary school students aged 13–20<br><br>*Programme Components:*<br>• Training peer educators to implement intervention in their schools<br>• HIV/AIDS education and awareness activities<br>• Class discussions of risk behaviours and gender relationships<br>• Provision of social support in solving personal problems	*Statistically Significant Effects:*<br>• Increase in the percentage of students in the control group who were sexually experienced	*Statistically Significant Effects:*<br>• Boys were more likely than girls to report being sexually active

Table 3: **Maternal & child health**

Programme	Programme Description	Illustrative Evidence - Health	Illustrative Evidence - Gender
**Boxgirls Afterschool Leadership Education**<br><br>Petry et al. 2017<br><br>*South Africa*	*Goal:* to enable girls to protect themselves from GBV through life skills, self-defence techniques, education, and leadership<br><br>*Participants:* girls aged 10–14 attending primary school<br><br>*Programme Components:*<br>• 8-week afterschool curriculum that uses games, discussion groups, community events, and homework support to cover:<br>• Communication skills<br>• Self-defence & exercise<br>• Self-respect & goal setting<br>• Personal safety<br>• Community mapping<br>• De-escalation strategies<br>• Local resources<br>• Girls' rights	*Statistically Significant Effects:*<br>• Increased body image scores	*Statistically Significant Effects:*<br>• Increases in global self-esteem scores<br>• Confidence in being able to defend oneself against an authoritative figure<br>• Positive change in school performance<br><br>*Non-significant Effects:*<br>• Report violence perpetrated by a loved one<br>• Family or friend self-esteem scores<br>• Family or friend support<br>• Sense of belonging<br><br>*Qualitative Findings:*<br>• Likelihood of wanting to and knowing how to intervene in the case of a friend being abused/experiencing violence<br>• Belief that performing well in school was important to achieving career goals and improving quality of life<br>• Ability to identify dangerous places and situations within one's community<br>• Ability to mention strategies on how to avoid or protect oneself in dangerous situations

Table 4: **Violence**

Programme	Programme Description	Illustrative Evidence - Health	Illustrative Evidence - Gender
**Coaching Boys into Men**<br><br>Miller at al.<br>2013<br><br>*United States (California)*	*Goal:* to reduce relationship violence among boys through altering gender norms that are supportive of IPV with messaging delivered by adult role models<br><br>*Participants:* high school athletic coaches, male athletes in grades 9–11<br><br>*Programme Components:*<br>• 60-minute training for coaches on the programme and provision of the Coaches Kit, which contained the curriculum and resources for programme delivery<br>• Facilitation of twelve 15-minute scripted lessons over the season that cover topics such as respect, nonviolence, sexual consent, and bystander intervention	*Statistically Significant Effects:*<br>• Reduced likelihood of perpetrating dating violence	*Statistically Significant Effects:*<br>• Lower levels of negative bystander behaviours<br><br>*Non-significant Effects:*<br>• Intentions to intervene<br>• Gender-equitable attitudes<br>• Recognition of abusive behaviours<br>• Positive bystander behaviours
**Coaching Boys into Men**<br><br>Jaime et al.<br>2015<br><br>*United States (California)*		*Statistically Significant Effects:*<br>• Number of incidents of unwelcomed actions towards girls (eg, sexual gestures, whistling) witnessed<br><br>*Non-significant Effects:*<br>• Number of abusive behaviours towards girls witnessed	*Statistically Significant Effects:*<br>• Increased bystander intervention behaviours<br>• Confidence in addressing athletes' abusive behaviours towards girls<br>• Discussions with athletes and other coaches about sexual violence and IPV<br><br>*Non-significant Effects:*<br>• Gender attitudes<br><br>*Qualitative Findings:*<br>• Observation of changes in athletes' attitudes and behaviours, particularly the use of more respectful language and behaviours towards peers<br>• Fear of retaliation for intervening
**Creating Opportunities through Mentorship, Parental Involvement, and Safe Spaces (COMPASS)**<br><br>Stark et al.<br>2018<br><br>*Democratic Republic of Congo (South Kivu)*	*Goal:* to prevent violence against girls through life skills, safe spaces, and promoting gender-equitable attitudes among caregivers<br><br>*Participants:* girls aged 10-14, their caregivers, women aged 18-30<br><br>*Programme Components:*<br>• Identification and training of female mentors<br>• "Adolescent Girl Safe Spaces" and life skills curriculum<br>• Thirty-two weekly meetings facilitated by female mentors covering various topics, including conflict resolution, decision-making, gender equitable norms, reproductive health, and safety planning<br>• Life skills training*<br>• Opportunity to develop social networks and mentor-mentee relationship<br>• Caregivers' discussion groups	*Non-significant Effects:*<br>• Engagement in transactional sex<br>• Physical, sexual, and emotional violence	*Statistically Significant Effects:*<br>• Increased caregiver warmth and affection and decreased rejection<br><br>*Non-significant Effects:*<br>• Experiences of all forms of violence<br>• Child marriage<br>• Neglect<br>• Caregivers' gender-equitable attitudes<br>• Caregivers' acceptance of physical discipline for children

Programme	Programme Description	Illustrative Evidence - Health	Illustrative Evidence - Gender
	• Thirteen monthly meetings during which facilitators deliver a structured curriculum on supporting girls, positive caregiver-girl relationships, non-violent discipline, and gender-equitable norms<br>• Caregivers discuss challenges to raising adolescent girls<br><br>**Received by girls in both intervention and control groups*		
**Creating Opportunities through Mentorship, Parental Involvement, and Safe Spaces (COMPASS)**<br><br>Stark et al. 2018<br><br>*Ethiopia (Benishangul-Gumuz)*	*Goal:* to prevent violence against girls through life skills, safe spaces, and promoting gender-equitable attitudes among caregivers<br><br>*Participants:* South Sudanese girls aged 13-19 living in refugee camps, their caregivers,<br><br>*Programme Components:*<br>• Identification and training of female mentors<br>• "Adolescent Girl Safe Spaces" and life skills curriculum<br>• Thirty weekly meetings facilitated by female mentors during consisting of 45-60 minutes structured content and 30 minutes of unstructured time<br>• Sessions focused on:<br>• Improving knowledge of GBV and SRH<br>• Skill building (*e.g.*, communication skills)<br>• Developing friendships<br>• Caregivers' discussion groups<br>• Eight monthly session during which they discuss how to support girls, improve their understanding of violence and abuse, and build communication skills<br>• Save spaces available to all girls and women for unstructured activities in between group sessions	*Non-significant Effects:*<br>• Engagement in transactional sex<br>• Physical, sexual, and emotional violence	*Statistically Significant Effects:*<br>• Improved attitudes around rites of passage<br>• Increased social support<br>• Increased odds of believe that girls should not get married or have children before age 18<br>• Greater number of girls reported believe that girls should complete an additional year of school<br>• Increased odds of having a friend their own age<br>• Increased odds of having a non-family female adult they trusted<br><br>*Non-significant Effects:*<br>• Feelings of safety<br>• Experiences of all forms of violence<br>• Child marriage
**Do Kadam Barabari Ki Ore**<br><br>JeJeebhoy et al. 2017<br><br>*India (Bihar)*	*Goal:* to prevent violence against girls and women through challenging gender-inequitable norms<br><br>*Participants:* boys aged 13-21 who are members of youth clubs<br><br>*Programme Components:*<br>• Identification and training of peer mentors<br>• Life skills education<br>• Forty-two weekly 1-hour group life skills education session covering gender roles and attitudes, positive masculinity, forms of violence against girls and women, and respectful behaviour<br>• Activities include discussions, case studies, and role plays<br>• Cricket<br>• One-hour cricket coaching in conjunction with life skills session<br>• Community engagement<br>• Participants support events to promote gender-equitable attitudes among community members	*Non-significant Effects:*<br>• Increased self-reports of bystander intervention<br>• Decreased perpetration of non-contact GBV (*e.g.*, stalking)<br><br>*Qualitative Findings:*<br>• No longer teasing girls was attributed to programme participation	*Statistically Significant Effects:*<br>• More equitable gender roles and constructs of masculinity<br>• Increased rejection of norms that support men's control over women<br>• Increased number of boys reporting they would respect peers who act in non-traditional ways (*e.g.*, refusing to use physical violence against partners, walking away from a fight)<br>• Decreased acceptability of GBV<br><br>*Qualitative Findings:*<br>• Decreased acceptance of any form of violence<br>• Increased knowledge of legal protections for women who experience marital violence<br>• Regularly helping with household work

Programme	Programme Description	Illustrative Evidence - Health	Illustrative Evidence - Gender
**El Joven Noble**<br><br>Kelly et al.<br>2010<br><br>*United States (South Texas)*	*Goal:* to prevent interpersonal violence through developing a connection to a gender-equitable and nonviolent cultural identity<br><br>*Participants:* 3rd–5th grade elementary school students, community members, school district administrators, social workers<br><br>*Programme Components:*<br>• Training Familias en Acción Community Collaborative Council staff and volunteers to facilitate program<br>• 10 weekly after-school educational sessions covering acknowledgment, understanding, integration, and movement<br>• Sessions' content was informed by indigenous teachings, cultural constructs, and core values of respect, dignity, trust, and care and love	*Statistically Significant Effects:**<br>• Higher scores on nonviolence self-efficacy and 'Program Values 1' (ie, it is important for a person to keep their work, take responsibility for their actions, not hurt others, be a positive example, be understanding, and give support) at immediate post-test<br>• Nonviolence self-efficacy at 3-month follow-up<br><br>*Non-significant Effects:*<br>• Change in scores on all evaluations<br><br>**Effects: seen among students at high risk of violence*	*Statistically Significant Effects:*<br>• Gender was significant as a covariate for 'Program Values 2,' dealing with attitudes towards machismo (direction not identified)
**Gender Equity Movement in Schools (GEMS)**<br><br>Achyut et al.<br>2016<br><br>*India*	*Goal:* to prevent violence by recognising and challenging inequitable norms and behaviours<br><br>*Participants:* girls and boys aged 12–14, teachers, school staff<br><br>*Programme Components:*<br>• Facilitation of a conversation on gender with the school staff at the beginning of implementation<br>• 12-day gender training workshops for teachers<br>• 24 monthly participatory sessions for students on gender, violence, bodily changes, relationships, emotions, communication, and conflict resolution<br>• 2 school-based campaigns including poster making, essay writing, games, and races	*Statistically Significant Effects:*<br>• Among boys<br>• Increased action in case of physical violence (eg, reporting to a teacher, trying to stop perpetrator)<br>• Reduction in use of violence	*Statistically Significant Effects:*<br>• Among girls and boys<br>• Increase in mean attitudinal scores with respect to gender and violence<br>• Disapproval of peer-based violence<br>• Among girls<br>• Positive action in the event of emotional violence
**Mentors in Violence Prevention (MVP)**<br>Williams & Neville<br>2017<br><br>*Scotland*	*Goal:* to prevention GBV through peer-learning and mentorship that encourages non-violence bystander intervention<br><br>*Participants:* high school students aged 11-18<br><br>*Programme Components:*<br>• Group sessions during which role plays of realistic scenarios, discussions on the issues presented, and proposals of appropriate responses are facilitated by peer mentors<br>• Sessions frame GBV within structures of power and control and promote bystander intervention and disapproval of violence perpetration and non-intervention during acts of violence	*Qualitative Findings:*<br>• Increased knowledge of the various forms of violence<br>• Self-reports of intervening in conflicts	*Qualitative Findings:*<br>• Peer mentors became trusted sources of support outside of group sessions, however, lack of privacy was a barrier to seeking assistance<br>• More supportive attitudes towards intervention<br>Decreased acceptance of violence

Programme	Programme Description	Illustrative Evidence - Health	Illustrative Evidence - Gender
**Planning Ahead for Girls' Empowerment and Employability (PAGE)**<br><br>Nanda et al. 2017<br><br>*India (Delhi)*	*Goal:* to increase girls' educational attainment and long-term health and socioeconomic development by providing life skills for formal employment and encouraging the development of career goals<br><br>*Participants:* low-income girls aged 15–17 attending government schools, parents, teachers<br><br>*Programme Components:*<br>• 4-module curriculum taught in schools, covering:<br>• Self: discussions of self, gender, power, and patriarchy<br>• Efficacy: skill development to make decisions and recognise the effects of discrimination on their lives<br>• Resourcefulness: skill development for financial management, CV writing, and accessing technology<br>• Employability: introductions to various industries and sectors through practicums<br>• Meetings with parents to discuss the programme and review PAGE concepts<br>• Orientation sessions with teachers to enhance student interaction	*Statistically Significant Effects:*<br>• Decreased reports of discrimination and violence	*Statistically Significant Effects:*<br>• Increase in the proportion of girls who said they have a say in decisions about when to marry<br>• Measures of self-efficacy<br>• Attitudes towards gender equality, discrimination and violence, and employability<br>• Feelings of acceptance by peers and teachers<br><br>*Non-significant Effects:*<br>• Intention to continue schooling
**Shifting Boundaries**<br><br>Taylor et al. 2013<br><br>*United States (New York City)*	*Goal:* to prevent dating violence and sexual harassment among middle school students by addressing attitudes and norms surrounding violent behaviours<br><br>*Participants:* 6th- and 7th-grade students, school staff<br><br>*Programme Components*:*<br>• Classroom instruction<br>• 6 sessions covering laws and consequences of violence and sexual harassment, communicating boundaries, and bystander intervention taught by substance abuse prevention and intervention specialists<br>• Building-level intervention<br>• Placement of posters throughout schools to build awareness and reporting of violence and sexual harassment<br>• Creation of temporary building-based restraining orders (ie, 'Respecting Boundaries Agreement')<br>• Identification of violence and sexual harassment 'hotspots' by students and monitoring by school staff<br><br>**Components were implemented separately and together in 3 intervention arms; control arm did not receive any of the 3 interventions*	*Statistically Significant Effects:*<br>• Both components<br>• Lower frequency of peer sexual violence victimisation and perpetration<br>• Building-level only<br>• Decrease in peer sexual violence victimisation and perpetration<br>• Decrease in dating sexual violence victimisation<br>• Lower frequency of peer to peer sexual violence	*Statistically Significant Effects:*<br>• Building-level only<br>• Decrease in peer sexual victimisation<br>• Higher odds of reporting sexual harassment
**Start Strong: Building Healthy Teen Relationships**<br><br>Miller et al. 2015	*Goal:* to reduce teen dating violence (TDV) through promoting skills and attitudes supportive of healthy relationships<br><br>*Participants:* middle school students aged 11–14 years, influential adults<br><br>*Programme Components:*	*Statistically Significant Effects:*<br>• Interpersonal communication<br><br>*Non-significant Effects:*<br>• Intimate partner violence<br>• Gender-based violence	*Statistically Significant Effects:*<br>• Increased boy/girlfriend relationship satisfaction and support<br>• Decreased acceptance of teen dating violence<br>• Decreased acceptance of negative gender stereotypes than control group

Programme	Programme Description	Illustrative Evidence - Health	Illustrative Evidence - Gender
United States	• Implementation of a school-based TDV curriculum that focuses on middle school students' developmental needs<br>• Engaging key influencers (ie, parents, caregivers, teachers, mentors) to help girls and boys understand healthy relationships<br>• Using social marketing strategies to disseminate messages about TDV and healthy relationships on Facebook<br>• Policy and environmental change (eg, incorporating TDV into existing bullying and sexual harassment policies)		• Long-term lower levels of gender stereotypes
**True Love**<br><br>Sosa-Rubi et al. 2017<br><br>*Mexico*	*Goal:* to prevent TDV through skill development and challenging norms regarding gender roles, dating violence, and relationships<br><br>*Participants:* low-income high school students, school staff<br><br>*Programme Components:*<br>• School Climate Change (SCC)*<br>• Schoolyard activities for students that promote respect and equality and use of community resources<br>• Five 4-hour workshops for school staff to raise awareness of the consequences of dating violence and identify actions that will promote nonviolence<br>• Individual-Level Components (IL)<br>• Classroom-based curriculum delivered in sixteen 1-hour weekly sessions covering beliefs around gender and dating violence; communication, problem-solving, and coping skills; behaviours that foster nonviolence; and the consequences of dating violence<br>• Additional schoolyard activities that give students the opportunity to use the knowledge gained during the sessions (eg, identifying high-risk areas at schools, distributing flyers on sexual and reproductive rights)<br><br>**Received by both intervention and control groups*	*Statistically Significant Effects:*<br>• SCC+IL<br>• Less experience of psychological dating violence<br>• Less experience of physical dating violence<br>• Less perpetration of psychological dating violence	*Statistically Significant Effects:*<br>• Both intervention and control groups<br>• Less acceptance and justification of violence<br>• SCC+IL<br>• Lower scores on the acceptance of sexist attitudes index among girls<br>• Greater knowledge of institutions that provide support to address dating violence among boys
**'You Wouldn't Sober, You Shouldn't Drunk'**<br><br>Wood & Shukla 2017<br><br>*United Kingdom*	*Goal:* to reduce the acceptability of unwanted sexual attention from others when drunk by influencing social norms<br><br>*Participants:* girls and boys aged 18–24<br><br>*Intervention Components:*<br>• Multimedia campaign released in 16-week groupings over 3 years through cinema advertising, posters, Spotify commercials, YouTube videos, and social media postings	*Statistically Significant Effects:*<br>• Lower tolerance of unwanted sexual attention<br>• Negative views towards harmful drinking behaviour	*Statistically Significant Effects:*<br>• Less tolerance of unwanted sexual attention among females<br>• Interaction between gender and intervention exposure
**Intervention Research**<br><br>Davis et al. 2002	*Goal:* to prevent sexual assault on college campuses by raising awareness of the connection between inequitable gender norms and rape supportive-behaviours and changing attitudes and beliefs<br><br>*Participants:* male members of college fraternities	*Non-significant Effects:*<br>• Attitudes re: sexual violence<br>• Comprehension of consent at 6-week follow-up	*Statistically Significant Effects:*<br>• Lower rape myth acceptance at immediate follow-up<br><br>*Non-significant Effects:*<br>• Rape myth acceptance at 6-week follow-up

Programme	Programme Description	Illustrative Evidence - Health	Illustrative Evidence - Gender
United States	*Intervention Components:*<br>• One 90-min socialisation-focused group session during which:<br>• Contemporary movie and TV programme clips are shown to illustrate the connection between sex role socialisation and rape<br>• Consent, communication, and the legal ramifications of rape are discussed		• Attitudes towards women at 6-week follow-up
**Intervention Research**<br><br>Gidycz et al. 2011<br><br>*United States (Midwest Region)*	*Goal:* to prevent sexual assault perpetration through challenging norms that perpetuate violence against women and encouraging pro-social bystander behaviours<br><br>*Participants:* 1st-year male university students<br><br>*Programme Components:*<br>• The 1.5-hour extracurricular group session including bystander intervention training, discussions regarding consent, education on and correction of harmful social norms surrounding sexual assault, and empathy induction through debunking rape myths<br>• A 'booster session' 4 months after initial session to review lessons and provide participants the opportunity to discuss their experiences using what they had learned	*Statistically Significant Effects:*<br>• Fewer men perpetrated acts of sexual aggression<br>• Greater likelihood to label a scenario of sexual aggression as rape<br>• Men in both intervention and control groups with a history of sexual aggression perpetrated more acts of sexual aggression over the 4-month follow-up period	*Statistically Significant Effects:*<br>• Beliefs that other men would intervene<br>• Reduced association with sexually aggressive men<br>• Fewer feelings of reinforcement for engaging in sexually aggressive behaviour<br><br>*Non-significant Effects:*<br>• Acceptance of rape myths<br>• Negative attitudes towards women<br>• Beliefs that male friends would disapprove of aggressive behaviour<br>• Likelihood to intervene
**Intervention Research**<br><br>Taylor et al. 2010<br><br>*United States (Cleveland, Ohio)*	*Goal*: to prevent GBV and sexual harassment through addressing attitudes towards dating violence and skill building<br><br>*Participants:* middle school students aged 11–13<br><br>*Programme Components:*<br>• 2 curricula were implemented in different classrooms, each consisting of 5 weekly 40-minute lessons taught by an educator from a local sexual assault centre or a local teacher<br>• Participatory, interaction-based curriculum<br>• Discussions on setting boundaries in relationships, forming deliberate friendships and relationships, the intimacy, continuum, the determination of wanted vs. unwanted behaviours, and the role of bystander as intervener<br>• Didactic, law- and justice-based curriculum<br>• Discussions on laws, definitions, information, and data about assault and penalties<br>**Control group received standard health education without dating violence prevention material*	*Statistically Significant Effects:*<br>• Lower prevalence and frequency of sexual victimisation among those who received the participatory curriculum<br>• Among individuals who received the law and justice curriculum:<br>• Lower prevalence of nonsexual violence perpetration<br>• Higher scores on the attitudes measures reducing sexual harassment<br>• Disposition about own/others' personal space<br>• Higher percentage of right answers<br>• Higher response rates of violence perpetration	*Statistically Significant Effects:*<br>• Less likely to agree that girls were at fault in sexual harassment among participants who received both curriculums<br>• Report of inappropriate attributions of girls' fault in sexual harassment among those who received the participatory curriculum at 6-month follow-up

Table 5: **Multiple areas of health**

FP/SRH – Family Planning & Sexual and Reproductive Health
HIV – HIV/AIDS
ID- Infectious Disease
MCH – Maternal & Child Health
MH – Mental Health
N – Nutrition
SA – Substance Abuse
V – Violence
WASH – Water, Sanitation, & Hygiene

Programme	Outcomes	Programme Description	Illustrative Evidence - Health	Illustrative Evidence - Gender
**Addis Birhan**<br><br>Erulkar et al. 2011<br><br>*Ethiopia*	FP/SRH, HIV, MCH, SA, V	*Goal:* to reduce girls' social vulnerability through male engagement<br><br>*Participants:* married men living in rural and peri-urban areas<br><br>*Programme Components:*<br>• Training male community members to act of mentors and mobilise groups for married men<br>• Implementation of a 30-hour curriculum during weekly men's group meetings that:<br>• Provides information and promotes dialogue on gender, relationships, caring for children, drugs and alcohol, HIV/AIDS, SRH, and violence<br>• Encourages self-exploration and emotional expressions	*Non-significant Effects:*<br>• STI prevention<br>• Family planning methods and use<br>• Decision making<br>• SRH<br>• Birth knowledge and preparedness<br>• Intimate partner violence	*Statistically Significant Effects:*<br>• More likely to have discussed plans for the future and health issues in the family with their spouse<br>• More likely to have helped with housework and fetched water<br>• More likely to report joint discussions with wives on how to use land in the last 3 months
**Adolescent Girls Empowerment Programme (AGEP)**<br><br>Austrian et al. 2016<br><br>*Zambia*	FP/SRH, HIV, V	*Goal:* to decreases girls' risk and vulnerability through building girls' social, economic, and health assets<br><br>*Participants:* girls aged 10–19<br><br>*Programme Components:*<br>• Safe Spaces (SS)<br>• Groups of 20–30 girls meet weekly with a trained mentor who provides information and training on health, life skills, and financial topics<br>• Health Vouchers (HV)<br>• Training providers on delivering adolescent-friendly services<br>• Provision of HVs redeemable at select public and private healthcare providers for a package of health services including wellness exams and age-appropriate SRH services<br>• Savings Accounts (SA)<br>• Girls Dream SAs tailored to girls<br>• Programme assistance in opening SAs at the National Savings and Credit Bank	*Statistically Significant Effects:*<br>• Increased average score on contraceptive knowledge scale (SS, SS+HV)<br>• Increased average score on SRH knowledge scale (SS)<br>• Percentage used condom at first sex (SS+HV)	*Statistically Significant Effects:*<br>• Percentage of girls who have place in community where they feel safe to meet girlfriends (SS+HV)<br>• Average score on non-acceptability of intimate partner violence (SS)<br>• Average score on financial literacy scale (SS, SS+HV, and SS+HV+SA)<br>• Percentage who saved in the last year and percentage who currently have at least 20 kwacha saved (SS+HV+SA)<br>• Average reported income in the last year (SS)

Programme	Outcomes	Programme Description	Illustrative Evidence - Health	Illustrative Evidence - Gender
**CARE ECD**<br><br>Clacherty et al. 2016<br><br>*Mozambique (Inhambane Province)*	ID, MCH, MH, N	*Goal:* to improve development outcomes of children under 5 through parental education and support<br><br>*Participants:* vulnerable households with children aged 18–48 months<br><br>*Programme Components*:*<br>• Training<br>• Identification and training of volunteer community members to make home visits and be advisors on child health and development<br>• Home visits<br>• Vulnerable families are visited weekly by trained community volunteers to:<br>• Provide health information using visual guides<br>• Form supportive relationships with children's' caregivers<br>• Refer caregivers to local services (eg, hospitals)<br><br>**The PROSAN programme was implemented in both intervention and control communities at the same time as CARE ECD. PROSAN aimed to reduce vulnerability of poor and food-insecure households through capacity strengthening, economic empowerment, and gender and power inequality*	*Statistically Significant Effects:*<br>• Decreased likelihood of experiencing emotional distress<br>• Increased childhood immunisations<br>• Higher dietary diversity among children<br>• Increased number of children sleeping under mosquito nets<br><br>*Non-significant Effects:*<br>• Prevalence of underweight children<br>• Improvements in developmental milestones<br><br>*Qualitative Findings:*<br>• Improved ability to care for children as a result of reduced emotional distress<br>• Caregivers experienced barriers (eg, transportation) to bringing children to clinics for regular growth monitoring or when they are ill<br>• Consistent visits of community volunteers motivated caregivers to apply what they learned during home-visits	*Statistically Significant Effects:*<br>• Increased knowledge of available services<br><br>*Qualitative Findings:*<br>• Community volunteers became trusted persons, which improved young mothers' feelings of social support
**Fourth R: Skills for Youth Relationships**<br><br>Wolfe et al. 2009<br><br>*Canada*		*Goal:* to reduce physical dating violence (PDV) through an integrated educational curriculum on healthy relationships<br><br>*Participants:* 9th-grade students aged 14–15, teachers, parents<br><br>*Intervention Components:*<br>• Individual-level activities<br>• 21-lesson curriculum taught in sex-segregated classes, which cover healthy, nonviolent relationships, personal safety and injury prevention, healthy growth and sexuality, and substance abuse<br>• Emphasis placed on development of skills to promote positive strategies for dealing with pressure and conflict within and outside of dating relationships<br>• School-level activities<br>• Teacher trainings re: dating violence and healthy relationships<br>• Provision of information for parents during 9th grade orientation and with 4 newsletters<br>• Student-led 'safe school committees' to engage students and community members in violence prevention activities<br><br>**Activities in control schools targeted similar objectives, but without the use of training or materials*	*Statistically Significant Effects:*<br>• Decreased likelihood to perpetrate PDV among boys<br>• Increased condom use among sexually active boys<br><br>*Non-significant Effects:*<br>• Rates of perpetration of PDV among girls<br>• Engagement in peer physical violence (eg, arguments, threats of violence, hurting another with the intention to humiliate, slapping)<br>• Condom use among all students<br>• Substance use	*Statistically Significant Effects:*<br>• Decreased experiences of IPV

Programme	Outcomes	Programme Description	Illustrative Evidence - Health	Illustrative Evidence - Gender
**Gender Roles, Equality, and Transformations (GREAT)**<br><br>Dagadu et al. 2016<br><br>*Uganda*	FP/SRH, V	*Goal:* to reduce GBV and improve SRH outcomes through a social marketing campaign that promotes gender-equitable attitudes and behaviours<br><br>*Participants:* girls and boys aged 10–19, community leaders and mobilisers, general community members<br><br>*Programme Components:*<br>• Twice-weekly 50-minute serial radio drama aimed at foster interest in community change and cultural revitalisation<br>• Community Action Groups (CAG)<br>• 9 CAG per parish implement the community action cycle (ie, collective dialogue on desired community-level changes and action planning)<br>• SRH service linkages<br>• Training for existing Village Health Teams on gender-sensitive service provision and adolescent-friendly SRH services<br>• Toolkit<br>• Activities tailored for different populations (ie, Flipbooks, activity cards, radio discussion guide and a community game) are used to promote discussion and learning	*Statistically Significant Effects:*<br>• Increased communication with partner about FP in last 3 months<br>• Increased FP seeking behaviour<br>• Increased intention to use FP in the future among 15–19 year olds who were married or cohabitating<br>• Decreased offense at wife requesting condom use<br>• Decrease in perception of childbearing as sign of real womanhood	*Statistically Significant Effects:*<br>• Increased equitable partner-decision making<br>• Increase in household role sharing<br>• Increase in couple communication scores<br>• Decreased violent response to partner<br>• Increased male advocacy for sister's education to parents<br>• Decreased inappropriate touching reported by males<br>• Increased self-efficacy to seek help for inappropriate touching<br>• Increased sharing of household chores among females and males attending school
**Girls First**<br><br>Leventhal et al. 2016<br><br>*India (Bihar)*	MH, N, WASH	*Goal:* to improve girls' physical health and wellbeing through knowledge, skills, and social support<br><br>*Participants:* very poor girls (average age 13) enrolled in middle school<br><br>*Programme Components:*<br>• Resilience Curriculum<br>• 23 weekly sessions that improve girls' psychosocial resilience, with a focus on goals and planning, social support, and identity<br>• Health Curriculum<br>• 21 weekly sessions aiming to improve knowledge, behaviours, and attitudes related to physical health issues through in-depth discussions and activities on SRH, nutrition, gender equality, and substance use<br><br>**Comparisons made to individual curriculums and standard school curriculum (control group)*	*Statistically Significant Effects:*<br>• Health curriculum<br>• Increased health knowledge<br>• Increased clean water behaviours<br>• Increased menstrual hygiene<br>• Increased health communication<br>• Decreased substance use<br>• Increased safety<br>• Increased vitality and functioning<br>• Resilience curriculum<br>• Increased gender equality attitudes<br>• Increased hand washing<br>• Increased menstrual hygiene<br>• Increased health communication<br>• Ability to safely get to doctor when needed<br>• Combined curriculums<br>• Increased health knowledge<br>• Increased gender equality attitudes<br>• Increased clean water behaviours<br>• Increased hand washing<br>• Increased health communication<br>• Ability to get to a doctor when needed<br>• Increased nutrition<br>• Increased safety	*Statistically Significant Effects:*<br>• Improved gender equality attitudes among girls who received the combined curriculum and the resilience only curriculum versus the standard school curriculum

Programme	Outcomes	Programme Description	Illustrative Evidence - Health	Illustrative Evidence - Gender
**HIV Prevention Trial Network 068 Study**<br>Kilburn et al.<br>2018<br><br>*South Africa (Mpumalanga Province)*	FP, V	*Goal:* to reduce IPV among girls through economic empowerment<br><br>*Participants:* poor, rural households with unmarried girls aged 13-20 enrolled in high school<br><br>*Programme Components:*<br>• Monthly cash transfers to girls of 100 Rand and 200 Rand to their parents or guardian conditional on attending at least 80% of school days during the month	*Statistically Significant Effects:*<br>• Delayed sexual debut<br>• Decreased number of sexual partners within the past year	*Statistically Significant Effects:*<br>• Decreased likelihood of experiencing physical IPV<br><br>*Non-significant Effects:*<br>• Decreased experiences of forced sex<br>• Improved perceptions of relationship power<br>Per capita household expenditure
**Involving Young Men to End Gender Violence**<br><br>Promundo & International Center for Research on Women<br>2012<br><br>*Chile*	FP/SRH, SA, V	*Goal:* to prevent GBV through male engagement<br><br>*Participants:* boys aged 14–19 living in low- and medium-income urban areas, government health professionals<br><br>*Programme Components:**<br>• Training<br>• 4-day training for government health professionals on how to work with youth and conduct community workshops with boys, which includes information on gender construction, masculinity, and multiple types of violence (eg, familial, IPV, assault, sexual abuse)<br>• Educational workshops<br>• 20 weekly workshops facilitated by trained health professionals in schools, community centres, and health centres<br>• Workshops focus on gender equity, masculinity, types of violence, alternatives to violence, and prevention<br><br>**Based on Program H*	*Statistically Significant Effects:*<br>• Increase in weapons use among men in control groups<br><br>*Non-significant Effects:*<br>• Condom use<br>• Alcohol consumption<br>• Increase in weapons use among men in intervention groups	*Statistically Significant Effects:*<br>• Increased joint decision making regarding condom use<br>• Increased attitudes against violence<br>• Negative correlation between condom use and acceptance of aggression towards others and IPV against a female partner<br><br>*Non-significant Effects:*<br>• Improved partner relationship<br>• Increased gender-equitable attitudes<br><br>*Qualitative Findings:*<br>• Increased understanding of types of violence and how to respond to aggression without violence<br>• Improved self-confidence<br>• Increased respect for people who differed from themselves
**Ishraq Program**<br><br>Brady et al.<br>2007<br><br>*Egypt*	FP/SRH, N	*Goal:* to empower girls in socially conservative contexts through education and gender norms change<br><br>*Participants:* out-of-school girls aged 13–15, parents, boys, community leaders<br><br>*Intervention Components:*<br>• Creation of safe spaces for girls<br>• Groups of 25 girls meet 4 times per week for 30 months to meet, learn, and play. Meetings include:<br>• Learn to Be Free literacy curriculum<br>• New Horizons curriculum to raise girls' awareness of self, family, health, and social issues<br>• Sports and physical activity	*Statistically Significant Effects:*<br>• Decrease in the percentage of participants reporting that they intend to circumcise their daughters in future	*Statistically Significant Effects:*<br>• Decrease in the percentage of girls reporting a preference to be married before age 18<br>• Increase in the percentage of girls reporting a desire for fewer than 3 children<br>• Increase in the mean scores on the gender role attitude index

Programme	Outcomes	Programme Description	Illustrative Evidence - Health	Illustrative Evidence - Gender
		• Home and vocational skills training including electrical appliance management and repair, hairdressing, sweets production<br>• Selection of 'Promoters' who facilitate girls' meetings and serve as intermediaries between the Ishraq participants and the community<br>• Assisting girls in obtaining identification cards and health insurance<br>• Creation of village committees that facilitate problem solving<br>• Family engagement<br>• New Visions curriculum on gender equity for girls' brothers<br>• Meetings with parents at youth centres to discuss their concerns and develop solutions		
**Kenya Cash Transfer for Orphans and Vulnerable Children**<br><br>Handa et al. 2015<br><br>*Kenya*	FP/SRH, MCH	*Goal:* to promote girls' safe transition into adulthood (ie, delayed marriage and pregnancy) through increased household economic wellbeing and investment in education<br><br>*Participants:* very poor households with at least 1 orphan or vulnerable child under 18 (ie, 1 or both parents deceased and/or primary caregiver with chronic illness)<br><br>*Programme Components:*<br>• Unconditional monthly cash transfer of 1 500 KSH (adjusted for inflation in 2011 to 2 000 KSH)	*Statistically Significant Effects:*<br>• Decreased likelihood of having ever been pregnant among girls	*Non-significant Effects:*<br>• Likelihood of early marriage
**Male Involvement Program (MIP)**<br><br>Brindis et al. 2005<br><br>*United States (California)*	FP/SRH, HIV, MCH	*Goal:* to prevent teenage pregnancy by encouraging boys' involvement in family planning education and services, challenging gender norms, and promoting safe transition to adulthood<br><br>*Participants:* low-income boys and men at risk of early fatherhood<br><br>*Programme Components:*<br>• While the length, magnitude, and type of programme varied across implementing agencies, each included:<br>• Community awareness of male involvement<br>• Community mobilisation<br>• Prevention education services<br>• Youth leadership development<br>• Youth-adult partnerships<br>• Institutionalisation of male involvement programmes<br>• Referral and linkages with clinical services	*Statistically Significant Effects:*<br>• Increased knowledge of risk of pregnancy<br><br>*Non-significant Effects:*<br>• Increased knowledge of risk of pregnancy using withdrawal<br>• Increased knowledge of where to find birth control<br>• Increased awareness of California's statutory rape law	*Statistically Significant Effects:*<br>Decreased number of participants reporting they would be happy if their partner became pregnant

Programme	Outcomes	Programme Description	Illustrative Evidence - Health	Illustrative Evidence - Gender
**Male Norms Initiative**<br><br>Pulerwitz et al. 2010<br><br>*Ethiopia (Addis Ababa)*	HIV, V	*Goal:* to prevent HIV, STI, and violence risk by challenging harmful gender norms and promoting gender equity<br><br>*Participants:* boys aged 15–24 who are members of a youth group, PEPFAR partner NGOs, PMTCT providers<br><br>*Programme Components:**<br>• Group education (GE)**<br>• 19 weekly 2-hour group education sessions that address gender norms and their relation to HIV prevention through role plays, groups discussions, and personal reflections<br>• Community engagement (CE)<br>• Community engagement activities to raise awareness and encourage dialogue including leaflets, newsletters, music, skits, community discussions, condom distribution, and a march for International Father's Day<br>• Capacity building and strengthening and technical assistance to enable PEPFAR partner NGOs and PMTCT providers to offer male engagement activities and promote constructive male engagement through existing programmatic work<br><br>**Based on Program H*<br>***Received by both intervention groups and by control group after 6 months*	*Statistically Significant Effects:*<br>• Decreased incidents of physical violence against a female partner<br><br>*Quantitative Findings:*<br>• Improved condom negotiation skills<br>• Reduced sexual risk behaviours<br>• Feeling better equipped to address gender in work	*Statistically Significant Effects:*<br>• Increased support of equitable gender norms<br>• Decreased support of inequitable norms<br><br>*Qualitative Findings:*<br>• Feeling more knowledgeable of gender issues<br>• Increased ability to communicate with their partners about HIV risk after<br>• Female partners indicated positive changes in their partners' behaviour, including communication regarding HIV and sex, helping with domestic chores, and reducing unhealthy behaviours
**Mpondombili**<br><br>Harrison et al. 2016<br><br>*South Africa (KwaZulu-Natal)*	FP/SRH, HIV	*Goal:* to prevent pregnancy and HIV through consistent condom use, delayed sexual debut, and gender norms supportive of preventive behaviours<br><br>*Participants:* girls and boys aged 14–17 enrolled in school, teachers<br><br>*Programme Components*:*<br>• Identification and training of adult role models and youth peer educators on curriculum content, gender norms, intergenerational relationships, participatory methods, and skills building<br>• Classroom-based curriculum for students<br>• Teachers and youth peer educators facilitate a 4-month intervention during the schools' guidance period<br>• 15 lessons cover a variety of topics including gender-related attitudes and social norms; self-efficacy and communication and negotiation skills; and HIV/AIDS and pregnancy prevention knowledge, attitudes, and behaviours<br>• Messages about 'dual protection' (ie, use of contraceptive methods that provide protection against both HIV and pregnancy) are integrated throughout each session<br>**Control group received the standard government-sponsored sexuality education course*	*Statistically Significant Effects:*<br>• Increase partner communication about condom among girls<br><br>*Non-significant Effects:*<br>• Condom use at last intercourse	*Statistically Significant Effects:*<br>• Decreased belief that condom use symbolises promiscuity<br>Increased self-efficacy for condom use<br>• Increased self-efficacy for refusing unsafe sex among boys and girls combined<br><br>*Non-significant Effects:*<br>• Condom use norms<br>• Gender beliefs about refusal of unsafe sex

Programme	Outcomes	Programme Description	Illustrative Evidence - Health	Illustrative Evidence - Gender
**The PRACHAR Project**<br><br>Daniel & Nanda 2012<br><br>*India (Bihar)*	FP/SRH, MCH	*Goal:* to increase girls' age at marriage, delay first birth after marriage, and ensure birth spacing through through awareness, knowledge, and understanding of reproductive health issues<br><br>*Participants:* girls and boys aged 15–19, young couples, their guardians (parents and in-laws), influential community members, local NGO staff<br><br>*Programme Components:*<br>• 3-day adolescent SRH education for girls and boys, covering a variety of topics including reproduction, menstruation, contraception, nutrition, STI transmission, gender equality, and egalitarian decision making<br>• Infotainment parties for newlywed couples<br>• Home visits for married women during which female health workers discuss the benefits of delaying birth and birth spacing<br>• Meetings with male spouses, guardians, and influential community members<br>• Dissemination of RH information in communities through wall paintings, posters, leaflets, and street theatre<br>• Community mobilisation activities to foster support for programme concepts	*Statistically Significant Effects:*<br>• Increased odds of contraception use after marriage and before first birth among women<br>• Increased odds of contraception use after marriage among married men<br>• Age at first birth for women was 1·5 years higher<br>• Decreased odds of having had a birth by the time of the 5-year follow-up	*Statistically Significant Effects:*<br>• Decreased odds (0·56) of women being married at 5-year follow-up ($p<0·001$)<br>• Decreased odds (0·74) of men being married at 5-year follow-up ($p<0·05$)<br>• Increased educational attainment among women ($p<0·05$) and men ($p<0·001$)<br><br>*Non-significant Effects:*<br>• Increased odds that women and men expressed to their parents the age at which they wanted to marry*<br><br>**p-value not reported*
**PRAGYA**<br><br>Pathfinder 2011<br><br>*India (Bihar)*	FP/SRH	*Goal: Retrospective analysis of* **PRACHAR** *Phase I and II*<br><br>*Participants:* girls and boys aged 12–24, young couples, their guardians (parents and in-laws), influential community members, local NGO staff	*Statistically Significant Effects:*<br>• Increased contraception use among couples who were both exposed to programme communications<br>• Increased SRH-related gender knowledge scores	*Statistically Significant Effects:*<br>• Increase in wife's participation in decision making related to contraceptive use<br>• Increased likelihood of delayed marriage<br>• Older age at marriage and first birth
**PREPARE**<br><br>Mathews et al. 2016<br><br>*South Africa (Cape Town)*	HIV, V	*Goal:* to prevent HIV through delaying sexual debut, increasing condom use, and reducing IPV<br><br>*Participants:* girls and boys (average age 13) enrolled in high school<br><br>*Programme Components:*<br>• Interactive, skills-based after-school education programme<br>• 21 weekly sessions covering assertive communication, intimate relationships, gender power inequalities, relationships, sexual decision-making, IPV, and HIV prevention<br>• School health service<br>• Weekly provision of free health services (eg, SRH education & referrals) delivered by nurses from local public clinics<br>• School sexual violence prevention programme<br>• 2-day training for School Safety Teams<br>• Photovoice project for select students	*Statistically Significant Effects:*<br>• Increased knowledge about condoms<br>• Increased knowledge about HIV/AIDS<br><br>*Non-significant Effects:*<br>• Sexual debut<br>• Self-reported condom use at last sex<br>• Number of sexual partners 6 months and 1 year from baseline	*Statistically Significant Effects:*<br>• Increased positive attitudes towards delaying sex<br>• Decreased IPV victimisation

Programme	Outcomes	Programme Description	Illustrative Evidence - Health	Illustrative Evidence - Gender
**Program H**<br><br>Pulerwitz et al. 2006<br><br>*Brazil (Rio de Janeiro)*	FP/SRH, HIV	*Goal:* to reduce HIV and STI risk by challenging gender norms around masculinity, reflecting on the consequences of inequitable gender norms, and promoting gender-equitable behaviours<br><br>*Participants:* low-income, in- and out-of-school boys aged 14–25<br><br>*Programme Components:*<br>• Two Components were implemented, each using gender-equitable messages to promote attitude, behaviour, and social norm changes about what it means to be a man<br>• Group Education*<br>• Weekly 2-hour sessions that cover 5 themes over the course of 6 months: sexuality and reproductive health; fatherhood and caregiving; from violence to peaceful coexistence; reasons and emotions (eg, communication skills, substance abuse, and mental health); preventing and living with HIV/AIDS<br>• Community-wide lifestyle social marketing campaign<br>• Messages reinforce concepts from the group education, promote gender equity, and encourage HIV, STI, and violence prevention at the community level<br>• 'Peer promoters' develop and implement messages and strategies to promote condom use as part of a more gender-equitable lifestyle<br><br>**Received by both intervention groups and by control group after 6 months*	*Statistically Significant Effects:*<br>• Increased condom use at last sex with a primary partner at 6-month follow-up among boys in both intervention arms and maintained through 12 months at the combined site<br>• Decreased reported STI symptoms at the combined site at 6-month follow-up and maintained through 12 months<br><br>*Non-significant Effects:*<br>• Condom use increased with casual partners at all 3 sites<br>• Reported STI symptoms at the group education-only site	*Statistically Significant Effects:*<br>• Decrease in the proportion of respondents who supported inequitable gender norms maintained through 12-month follow-up at both intervention sites<br><br>*Non-significant Effects:*<br>• Partner communication about HIV and condoms increased<br><br>*Qualitative Findings:*<br>• Group education sessions served as safe spaces for boys to discuss issues not typically spoken about (eg, community violence, relationships, family life)
**Saloni Pilot Program**<br><br>Kapadia-Kundu et al. 2014<br><br>*India (Uttar Pradesh)*	FP/SRH, N, WASH	*Goal:* to break the cycle of intergenerational ill-health by changing health behaviours in girls related to anaemia, maternal mortality, and under-nutrition<br><br>*Participants:* girls aged 11–14 enrolled in government schools<br><br>*Programme Components:*<br>• School-based educational curriculum<br>• Ten 1-hour health education sessions delivered monthly by teachers covering nutrition, health-seeking behaviours, reproductive health, and hygiene<br>• Intergenerational communication is stressed throughout each session. Role plays encourage girls to start conversations with their parents about what they learned, participants practiced during the sessions through role plays<br>• Saloni Diaries<br>• Diaries that provide girls with activities, systems to track health behaviours, and document life goals	*Statistically Significant Effects:*<br>• Increased consumptions of weekly iron and folic acid (IFA) tablets<br>• Increased DOTS for girls who consume IFA<br>• Attendance at annual school health check-up<br>• Increased consumption of deworming tablet every 6 months<br>• Increased consumption of at least 3 meals + snack per day<br>• Increase in food variety index >8<br>• Increased consumption of sprouted legumes<br>• Increased consumption foods with vitamin C<br>• Increased daily genital hygiene<br>• Increased changing of menstruation cloth 3×/day<br>• Increased belief that ideal number of children is <2<br>• Increased daily hand washing with soap after defecation, before cooking, and before a meal	*Statistically Significant Effects:*<br>• Intergenerational communication with parents on programme health topics<br>• Increased access to and use of services

Programme	Outcomes	Programme Description	Illustrative Evidence - Health	Illustrative Evidence - Gender
**Shaping the Health of Adolescents in Zimbabwe Project (SHAZ!)**<br><br>Dunbar et al. 2014<br><br>*Zimbabwe (Harare)*	FP/SRH, HIV	*Goal:* to prevent HIV infection among girls by addressing structural barriers<br><br>*Participants:* out-of-school girls aged 16–19 who had lost at least 1 parent<br><br>*Programme Components:*<br>• SRH services, including health screenings, STI treatment, provision of free contraception, and referrals for HIV treatment*<br>• Life skills and home-based care training*<br>◦ 14-module life skills curriculum delivered over 46 weeks, covering a variety of topics including SRH, relationship negotiation, and strategies to avoid violence<br>◦ 6-week course on how to safely care for people living with HIV/AIDS<br>• Livelihoods training including courses on financial literacy, vocational training, and US$100 micro-grants for completed business plans<br>• Integrated Social Support through guidance counselling provided by trained staff to help participants navigate challenges and adult mentorship<br><br>**Received by both intervention and control groups*	*Statistically Significant Effects:*<br>• Lower food insecurity<br><br>*Non-significant Effects:*<br>• Reduced risk of transactional sex<br>• Higher likelihood of using a condom with their current partner<br>• Fewer unintended pregnancies<br>• HIV incidence	*Statistically Significant Effects:*<br>• Increased odds of having their own income<br><br>*Non-significant Effects:*<br>• Reduced experience of violence over time
**Strengthening Household Ability to Respond to Development Opportunities project (SHOUHARDO)**<br><br>Smith et al. 2011<br><br>*Bangladesh*	MCH, N, WASH	*Goal:* to reduce child malnutrition, poverty, and food insecurity through addressing structural causes with a rights-based, livelihoods approach<br><br>*Participants:* poor households with children aged 6–24 months<br><br>*Programme Components:*<br>• Mother and Child Health and Nutrition<br>◦ Food rations for children 6–24 months and pregnant and lactating mothers<br>◦ Health and nutrition education through mothers' group programmes<br>◦ Child growth monitoring<br>◦ Provision of prenatal care and birth planning<br>◦ Aid in obtaining emergency obstetric care for mothers<br>◦ Vitamin A supplementation for children and Vitamin A and iron-folic acid supplementation for mothers<br>◦ Immunisations<br>◦ Referrals for family planning and emergencies; and<br>◦ Facilitation of access to local health facilities<br>• Sanitation<br>◦ Installation of tube wells to obtain arsenic-free water	*Statistically Significant Effects:*<br>• Increased food security<br>• Increased care practices for children 6–24 months, including children fully immunised before 1, Vitamin A supplementation, ORT during diarrhoea, and breastfeeding<br>• Increased care practices for mothers, including 3 antenatal visits, iron/folic acid/vitamin A supplementation, food intake during pregnancy, and daytime rest<br>• Increased access to safe water and sanitary latrine<br><br>*Non-significant Effects:*<br>• 15·7% decrease in stunting prevalence<br>• Increase in stunting among children 6–18 months old and children 48–60 months old	*Statistically Significant Effects:*<br>• The women's empowerment activities had an independent impact on stunting<br>• Synergistic effects between MNCH activities on stunting:<br>◦ Women's empowerment activities<br>◦ Sanitation activities<br>◦ Poverty alleviation activities

Programme	Outcomes	Programme Description	Illustrative Evidence - Health	Illustrative Evidence - Gender
		• Provision of arsenic testing<br>• Increasing access to latrines<br>• Women's Empowerment<br>• Creation of groups for women and their adolescent daughters<br>• Provision of early child care for development (ECCD) for girls<br>• Formation of Parent Teacher Associations to facilitate participation of women in schools and education of daughters<br>• Poverty and Food Security Alleviation<br>• Training and inputs to promote field crop and fisheries production, homestead gardening and livestock rearing, and cash income-generating activities<br>• Development of food-for-work and cash-for-work projects that included infrastructure development<br>• Establishment of savings groups to help households pool financial resources<br>• Empowerment of the Poor<br>• Creation of Village Development Committees<br>• Capacity building for local response to disasters		
**Si yo estoy bien, mi familia también**<br><br>Venguer et al. 2007<br><br>*Mexico (Oaxaca)*	FP/SRH, MCH, N, WASH	*Goal:* to improve the intergenerational health of girls and their children through education, increased agency, and life skills building<br><br>*Participants:* girls aged 12–20 living in rural areas, various community groups<br><br>*Programme Components:*<br>• Training<br>• Initial training for community action promoters (CAP) on programme messages, group management, and use of didactic materials (eg, videos, pamphlets, flash cards, flip charts)<br>• Replication of training by CAPs for rural health assistants, who in turn train social volunteer promoters<br>• Educational programme<br>• Social volunteer promoters provide the 4-module curriculum to groups of 10–15 women. Topics covered include health and agency (eg, right to health, decision making, communication), nutrition, hygiene, and SRH<br>• Advocacy with authorities (eg, government officials, medical staff, school teachers)<br>• Dissemination of programme messages to general community members through public and media events, distribution of promotional materials (eg, posters and pamphlets), and video presentations	*Statistically Significant Effects:*<br>• Increased likelihood of contraception use<br>• Increase knowledge on STI prevention<br>• Increased likelihood of having obtained a Pap test	*Statistically Significant Effects:*<br>• Women more likely to defend their own opinions

Programme	Outcomes	Programme Description	Illustrative Evidence - Health	Illustrative Evidence - Gender
**SKILLZ Street**<br>Merrill et al. 2018<br><br>*South Africa (Soweto)*	FP/SRH, HIV, V	*Goal:* to reduce girls' risk for HIV and violence and improve their SRH outcomes through developing life skills<br><br>*Participants:* girls aged 10-14 enrolled in primary school, female community members 18-26<br><br>*Programme Components:*<br>• Eighty-hour training for female community members to become SKILLZ Street coaches and on the "Coach Tumi" text-message service<br>• After-school program<br>◦ Ten, 2-hour after-school group sessions that included:<br>▪ Structured discussions and soccer-based life skills relating to body image, SRH knowledge, HIV, and decision-making in relationships<br>▪ Soccer games and activities<br>◦ Homework activities<br>◦ Graduation ceremony<br>• Two-way text messaging campaign ("Coach Tumi") that provide:<br>◦ Information on gender violence and community health services<br>◦ Quizzes on the programme, "girls topics", relationships, gender, and rights and responsibilities	*Non-significant Effects:*<br>• Small improvements in:<br>◦ HIV-related knowledge<br>◦ Knowledge of services for sexual violence<br>• Decreased knowledge of services for pregnancy prevention, abortion, and HIV testing<br><br>*Qualitative Findings:*<br>• Increased communication with parents, siblings, peers, and friends on HIV, SRH, and family planning	*Non-significant Effects:*<br>• Moderate improvements in:<br>◦ Self-esteem<br>◦ Self-efficacy to protect themselves against unwanted sex<br>◦ Perceptions of reaching a power balance in relationships<br>◦ Gender-equitable attitudes<br><br>*Qualitative Findings:*<br>• Teachers reported increased school attendance and performance among programme participants
**Stepping Stones**<br><br>Jewkes et al. 2008<br><br>*South Africa (Eastern Cape)*	FP/SRH, HIV, V, SA	*Goal*: to prevent HIV and improve SRH through promoting gender-equitable relationships<br><br>*Participants:* Girls and boys aged 15–26, NGO staff, community members (programme's intended ages: 16–23)<br><br>*Programme Components*:*<br>• 3-week training for Planned Parenthood Association of South Africa staff on programme implementation<br>• Single-sex group workshops<br>◦ A 50-hour, 13-session curriculum covering a multitude of topics including sex and love, contraception, STIs/HIV, GBV, communication skills, and motivations behind behaviours<br>◦ Strategies utilised include discussions, role plays, dramas, and critical reflection, in addition to the curriculum-based sessions<br>• 3 meetings between female and male peer groups<br>• Community meeting at culmination of programme<br><br>**Control groups received one 3-hour educational session on HIV, safe sex, and condom use. Content taken from the Stepping Stones South Africa curriculum*	*Statistically Significant Effects:*<br>• Decreased incidence of HSV-2 over 2 years<br>• Fewer boys self-reported problematic alcohol use at 1-year follow-up<br><br>*Non-significant Effects:*<br>HIV incidence<br>• Perpetration of sexual violence<br>• Number of partners in the past year Perpetration of rape or attempted rape pregnancy<br>• Correct condom use<br>• Depression<br>• Problematic alcohol use among girls<br>• Misuse of drugs	*Statistically Significant Effects:*<br>• Decreased engagement in transactional sex with a casual partner among girls at 1-year follow-up<br><br>*Non-significant Effects:*<br>• Experiences of physical or sexual IPV at 2-year follow-up among girls

Programme	Outcomes	Programme Description	Illustrative Evidence - Health	Illustrative Evidence - Gender
**TOSTAN**<br><br>Diop et al. 2004<br><br>*Senegal*	FP/SRH, WASH	*Goal:* to improve women's health and encourage the abandonment of FGC and forced child marriage through education and social mobilisation<br><br>*Participants:* women and men, community leaders, local government, civil society, NGO staff<br><br>*Programme Components:*<br>• Basic education programme with 4 modules: hygiene, problem solving, women's health, and human rights<br>• Discussions on programme activities with community leaders<br>• Capacity building with local NGO staff to implement programme<br>• Training local community members to be facilitators and supervisors<br>Public declaration renouncing FGC	*Statistically Significant Effects:*<br>• Increased use of antenatal services<br>Increased proportion of girls aged 0–10 yrs who had not been cut	*Statistically Significant Effects:*<br>• Increased awareness of human rights<br>• Decreased proportion of men intending to have their daughters cut decreased significantly*<br>• Decreased proportion of women reporting experiencing violence in the past 12 months<br><br>**Effects greatest among individuals directly exposed to programme*
**Yaari-Dosti**<br><br>Verma et al. 2008<br><br>*India (Mumbai & Gorakhpur)*	HIV, V	*Goal:* to reduce HIV risk and GBV through promoting gender-equitable norms and behaviours<br><br>*Participants:* boys and men aged 15–24 in rural area<br><br>*Programme Components:**<br>• Group education (GES)<br>• 23 weekly sessions covering a variety of topics including STI/HIV risk and prevention, substance use, sexuality, gender, power, and violence<br>• Strategies utilised include role plays, games, discussions, debates, and critical reflection<br>• Lifestyle social marketing campaign (LSSM)<br>• Campaign messages promote an alternate form of masculinity and focus on 4 themes:<br>• Nonviolent relationships<br>• Gender-equitable attitudes<br>• View of girls and women as human beings who deserve respect<br>• Shared responsibility in SRH and risk reduction<br>• Activities, including street plays, posters, pamphlets, comic strips, community discussions, and condom distribution, are held at places in the community where boys and men naturally gather<br><br>**Based on Program H*	*Statistically Significant Effects:*<br>• Increase in the percentage of men reporting partner communication about condoms, sex, STIs, and/or HIV<br>• GES participants<br>• More likely to report condom use at last sex<br>• Less likely to report committing partner violence<br>• More likely to report a positive change in attitudes towards people living with HIV	*Statistically Significant Effects:*<br>• GES+LSSM participants<br>• Positive improvement in disagreeing with traditional gender norms<br>• Negative changes in support of the following statements:<br>• It's okay for a man to hit his wife if she won't have sex with him<br>• There are times when a woman deserves to be beaten<br>• A man is happily married only if his wife brings a big dowry

Programme references

Achyut P, Bhatla N, Verma H, Singh G, Bhattacharya S, Verma R. Towards gender equality: the GEMS journey thus far. An evaluation report of the Gender Equity Movement in Schools (GEMS) program in Jharkhand. New Delhi: International Center for Research on Women (ICRW), 2016.

Amin S, Suran L. Program efforts to delay marriage through improved opportunities: some evidence from rural Bangladesh. Presented at the 2005 Annual Meeting of the Population Association of America, Philadelphia, Pennsylvania, March 31-April 2, 2005. Available from: https://www.popline.org/node/267355 New York: Population Council, 2005.

Austrian K, Hewett PC, Soler-Hampejsek E, Bozzani F, Behrman JR, Digitale J. Adolescent Girls Empowerment Programme: research and evaluation mid-term technical report. Lusaka, Zambia: Population Council, 2016.

Bandiera O, Buehren N, Burgess R, et al. Empowering adolescent girls: evidence from a randomized control trial in Uganda. Report number: 75566. London: London School of Economics, 2012.

Brady M, Assaad R, Ibrahim B, Salem A, Salem R, Zibani N. Providing new opportunities to adolescent girls in socially conservative settings: the Ishraq program in rural Upper Egypt. New York: Population Council, 2007.

Brindis CD, Barenbaum M, Sanchez-Flores H, McCarter V, Chand R. Let's hear it for the guys: California's Male Involvement Program. *Int J Mens Health* 2005; **4:** 29–53.

Clacherty G, Clacherty A, Tredoux C, Dawson M. ECD program impact evaluation report. CARE International, 2016.

Constantine NA, Jerman P, Berglas NF, Angulo-Olaiz F, Chou CP, Rohrbach LA. Short-term effects of a rights-based sexuality education curriculum for high-school students: a cluster-randomized trial. *BMC Public Health* 2015; **15:** 1–13.

Dagadu N, Lundgren R, Steven V, McCadden D, Savage S. GREAT project endline report. Washington, DC: Institute for Reproductive Health, Georgetown University; Pathfinder International; Save the Children; 2016.

Daniel EE, Nanda R. The effect of reproductive health communication interventions on age at marriage and first birth in rural Bihar, India: a retrospective study. Watertown, MA: Pathfinder International, 2012.

Davis T L, Liddell DL. Getting inside the house: the effectiveness of a rape prevention program for college fraternity men. *J Coll Stud Dev* 2002; **43:** 35–50.

Diop N, Faye MM, Moreau A, et al. The TOSTAN program: evaluation of a community based education program in Senegal. Population Council, 2004.

Dunbar MS, Kang Dufour MS, Lambdin B, Mudekunye-Mahaka I, Nhamo D, Padian NS. The SHAZ! project: results from a pilot randomized trial of a structural intervention to prevent HIV among adolescent women in Zimbabwe. *PLoS One* 2014; 9:

Erulkar AS, Chong E. *Evaluation of a Savings & Micro-Credit Program for Vulnerable Young Women in Nairobi.* Population Council. 2005.

Erulkar AS, Muthengi E. Evaluation of Berhane Hewan: A Program To Delay Child Marriage in Rural Ethiopia. *International Perspectives on Sexual and Reproductive Health.* 2009;35 (1): 6–14.

Erulkar A, Apicella L, Ferede A. *Addis Birhan Project: Working with Boys and Men to Address Young Girls' Social Vulnerability.* Population Council. Report number: 6, 2011.

Erulkar A, Ferede A, Girma W, Ambelu W. Evaluation of 'Biruh Tesfa' (Bright Future) Program for Vulnerable Girls in Ethiopia. *Vulnerable Children and Youth Studies*. 2013;8 (2): 182–92.

Gidycz CA, Orchowski LM, Berkowitz AD. Preventing Sexual Aggression Among College Men: An Evaluation of a Social Norms and Bystander Intervention Program. *Violence Against Women.* 2011;17 (6): 720–742.

Handa S, Peterman A, Huang C, Halpern C, Pettifor A, Thirumurthy H. Impact of the Kenya Cash Transfer for Orphans and Vulnerable Children on Early Pregnancy and Marriage of Adolescent Girls. *Social Science & Medicine.* 2015;141: 36–45.

Harper GW, Bangi AK, Sanchez B, Doll M, Pedraza A. A Quasi-Experimental Evaluation of a Community-Based HIV Prevention Intervention for Mexican American Female Adolescents: The SHERO's Program. *AIDS Education & Prevention.* 2009;21 (Supplement B): 109–123.

Harrison A, Hoffman S, Mantell JE, Smit JA, Leu CS, Exner TM, Stein ZA. Gender-Focused HIV and Pregnancy Prevention for School-Going Adolescents: The Mpondombili Pilot Intervention in KwaZulu-Natal, South Africa. *Journal of HIV/AIDS & Social Services*. 2016;15 (1): 29–47.

Institute for Reproductive Health at Georgetown University. *Transforming Gender Norms among Very Young Adolescents: An Innovative Intervention and Evaluation in Nepal.* 2013.

Jaime MCD, McCauley HL, Tancredi DJ, Nettiksimmons J, Decker MR, Silverman JG, O'Connor B, Stetkevich N, Miller E. Athletic Coaches as Violence Prevention Advocates. *Journal of Interpersonal Violence.* 2015;30 (7): 1090–1111.

Jejeebhoy SJ, Santhya K. Preventing violence against women and girls in Bihar: challenges for implementation and evaluation. Reproductive Health Matters. 2018;26(52):1470430.

Jewkes R, Nduna M, Levin J, Jama N, Dunkle K, Puren A, Duvvury N. Impact of Stepping Stones on Incidence of HIV and HSV-2 and Sexual Behaviour in Rural South Africa: Cluster Randomised Controlled Trial. *BMJ.* 2008;337:a506.

Kanesathasan A, Cardinal LJ, Pearson E, Gupta SD, Mukherjee S, Malhotra A. *Catalyzing Change: Improving Youth Sexual and Reproductive Health through Disha, an Integrated Program in India.* International Center for Research on Women. 2008.

Kapadia-Kundu N, Storey D, Safi B, Trivedi G, Tupe R, Narayana G. Seeds of Prevention: The Impact on Health Behaviors of Young Adolescent Girls in Uttar Pradesh, India, a Cluster Randomized Control Trial. *Social Science & Medicine.* 2014;120: 169–179.

Karim AM, Williams T, Patykewich L, Ali D, Colvin CE, Posner J, Rutaremwa G. The Impact of the African Youth Alliance Program on the Sexual Behavior of Young People in Uganda. *Studies in Family Planning.* 2009;40 (4): 289-306.

Kelly PJ, Lesser J, Cheng AL, Osóos-Sánchez M, Martinez E, Pineda D, Mancha J. A Prospective Randomized Controlled Trial of an Interpersonal Violence Prevention Program With a Mexican American Community. *Family & Community Health.* 2010;33 (3): 207–215.

Kilburn K, Hughes JP, Macphail C, Wagner RG, Gómez-Olivé FX, Kahn K, et al. Cash Transfers, Young Women's Economic Well-Being, and HIV Risk: Evidence from HPTN 068. AIDS and Behavior. 2018Oct;.

Leventhal KS, DeMaria LM, Gillham JE, Andrew G, Peabody J, Leventhal SM. A Psychosocial Resilience Curriculum Provides the 'Missing Piece' to Boost Adolescent Physical Health: A Randomized Controlled Trial of Girls First in India. *Social Science & Medicine.* 2016;161: 37–46.

Mathews C, Eggers SM, Townsend L, Aarø LE, de Vries PJ, Mason-Jones AJ, De Koker P, et al. Effects of PREPARE, a Multi-Component, School-Based HIV and Intimate Partner Violence (IPV) Prevention Programme on Adolescent Sexual Risk Behaviour and IPV: Cluster Randomised Controlled Trial. *AIDS and Behavior.* 2016;20(9): 1821–1840.

Maticka-Tyndale E, Wildish J, Gichuru M. Quasi-Experimental Evaluation of a National Primary School HIV Intervention in Kenya. *Evaluation and Program Planning.* 2007;30(2): 172–186.

Merrill KG, Merrill JC, Hershow RB, Barkley C, Rakosa B, Decelles J, et al. Linking at-risk South African girls to sexual violence and reproductive health services: A mixed-methods assessment of a soccer-based HIV prevention program and pilot SMS campaign. Evaluation and Program Planning. 2018;70:12–24.

Miller E, Tancredi DJ, McCauley HL, Decker MR, Virata MCD, Anderson HA, O'Connor B, Silverman JG. One-Year Follow-Up of a Coach-Delivered Dating Violence Prevention Program. *American Journal of Preventive Medicine.* 2013;45(1): 108–112.

Miller S, Williams J, Cutbush S, Gibbs D, Clinton-Sherrod M, Jones S. Evaluation of the Start Strong Initiative: Preventing Teen Dating Violence and Promoting Healthy Relationships Among Middle School Students. *Journal of Adolescent Health.* 2015;56 (2): S14–S19.

Nanda P, Gautam A, Das P, Vyas A, Guhathakurta A, Datta N. *Shaping Futures: Planning Head for Girls' Empowerment and Employability. An Evaluation Study of a School-Based Girls' Gender Integrated Skills Program in Delhi, India.* International Center for Research on Women. 2017.

Pathfinder International. *PRAGYA - Multisectoral, Gendered Approach to Improve Family Planning and Sexual and Reproductive Health for Young People: A Research Study.* Pathfinder International. 2011.

Petersen I, Bhagwanjee A, Bhana A, Mahintsho Z.The Development and Evaluation of a Manualised Participatory HIV/AIDS Risk Reduction Programme (Sex and Risk) for Tertiary Level Learners: A Pilot Study. *African Journal of AIDS Research.* 2004;3: 93–100.

Petry K, Bierman M, Gerka N, Maleske C. *Final Report: Boxgirls Afterschool Clubs Evaluation, January 2015 to April 2017.* Institute of European Sport Development and Leisure Studies. 2017.

Pham V, Nguyen H, Tho LH, Minh TT, Lerdboon P, Riel R, Green MS, Kaljee LM. Evaluation of Three Adolescent Sexual Health Programs in Ha Noi and Khanh Hoa Province, Vietnam. *AIDS Research and Treatment.* 2012;2012(986978): 1-12.

Promundo and Interntional Center for Research on Women. *Proyecto involucrando hombres jóvenes en el fin de la violencia de género: intervención multipaís con evaluación de impacto Informe final-caso Chileno.* Promundo. Proyecto coordinado a nivel global por Promundo, Brasil, y por el International Center for Research on Women (ICRW), Washington DC, Estados Unidos. 2012. Available from: https://www.researchgate.net/publication/282672444

Pulerwitz J, Barker G, Segundo M, Nascimento M. *Promoting More Gender-Equitable Norms and Behaviors Among Young Men as an HIV/AIDS Prevention Strategy.* Population Council. 2006.

Pulerwitz J, Martin S, Mehta M, Castillo T, Kidanu A, Verani F, Tewolde S. *Promoting Gender Equity for HIV and Violence Prevention: Results from the PEPFAR Male Norms Initiative Evaluation in Ethiopia.* PATH. 2010

Sebastian MP, Mensch B, Grant M. *Integrating Adolescent Livelihood Activities within a Reproductive Health Programme for Urban Slumdwellers in India.* Population Council. 2004.

Shahnaz R, Karim R. *Providing Microfinance and Social Space to Empower Adolescent Girls: An Evaluation of BRAC's ELA Centres.* BRAC Research Evaluation Decision. Report Number: 3, 2008.

Smith KV, Dye C, Rotz D. *Impact Report from the Evaluation of Adolescent Pregnancy Prevention Approaches.* U.S. Department of Health and Human Services, Office of Adolescent Health. 2016.

Smith LC, Kahn F, Frankenberger TR, Wadud A. *Admissible Evidence in the Court of Development Evaluation?: The Impact of CARE's SHOUHARDO Project on Child Stunting in Bangladesh.* Number: 376. Brighton: IDS; 2011. Available from: http://www.ids.ac.uk/files/dmfile/Wp376.pdf

Solórzano I, Bank A, Peña R, Ellsberg M, Pulerwitz J. *Catalyzing Personal and Social Change Around Gender, Sexuality, and HIV: Impact Evaluation of Puntos de Encuentro's Communication Strategy in Nicaragua.* Population Council. 2008.

Sosa-Rubi SG, Saavedra-Avendano B, Piras C, Van Buren SJ, Bautista-Arredondo S. True Love: Effectiveness of a School-Based Program to Reduce Dating Violence Among Adolescents in Mexico City. *Prevention Science.* 2017;18 (7): 804–817.

Ssewamala F, Ismayilova L, McKay M, Sperber E, Bannon W, Alicea S. Gender and the Effects of an Economic Empowerment Program on Attitudes Toward Sexual Risk-Taking Among AIDS-Orphaned Adolescent Youth in Uganda. *The Journal of Adolescent Health : Official Publication of the Society for Adolescent Medicine.* 2010;46 (4): 372–378.

Stark L, Seff I, Asghar K, Roth D, Bakamore T, Macrae M, et al. Building caregivers' emotional, parental and social support skills to prevent violence against adolescent girls: findings from a cluster randomised controlled trial in Democratic Republic of Congo. BMJ Global Health. 2018;3(5).

Stark L, Asghar K, Seff I, Yu G, Gessesse TT, Ward L, et al. Preventing violence against refugee adolescent girls: findings from a cluster randomised controlled trial in Ethiopia. BMJ Global Health. 2018;3(5).

Taylor BG, Stein ND, Mumford EA, Woods D. Shifting Boundaries: An Experimental Evaluation of a Dating Violence Prevention Program in Middle Schools. *Prevention Science*. 2013;14 (1): 64–76.

Taylor B, Stein N, Burden F. The Effects of Gender Violence/Harassment Prevention Programming in Middle Schools: A Randomized Experimental Evaluation. *Violence and Victims.* 2010;25 (2): 202–23.

Venguer T, Pick S, Fishbein M. Health Education and Agency: A Comprehensive Program for Young Women in the Mixteca Region of Mexico. *Psychology, Health & Medicine.* 2007;12 (4): 389–406.

Verma R, Pulerwitz J, Mahendra V, Khandekar S, Singh AK, Das SS, Mehra S, Nura A, Barker G. *Promoting Gender Equity as a Strategy to Reduce HIV Risk and Gender-Based Violence Among Young Men in India.* Population Council. 2008

Visser M. HIV/AIDS Prevention through Peer Education and Support in Secondary Schools in South Africa. *SAHARA-J: Journal of Social Aspects of HIV/AIDS.* 2007;4 (3): 678–694.

Williams DJ, Neville FG. Qualitative evaluation of the mentors in violence prevention pilot in Scottish high schools. Psychology of Violence. 2017;7(2):213–23.

Wolfe DA, Crooks C, Jaffe P, Chiodo D, Hughes R, Ellis W, Stitt L, Donner A. A School-Based Program to Prevent Adolescent Dating Violence: A Cluster Randomized Trial. *Archives of Pediatrics & Adolescent Medicine.* 2009;163 (8): 692–699.

Wood M, Shukla P. You Wouldn't Sober, You Shouldn't Drunk: A Behavioural Change Approach to Changing Attitudes and Responses to Unwanted Sexual Attention in Pubs and Clubs. *Alcohol and Alcoholism.* 2017;52 (6): 737–745.
